# Cell Type Specific Alterations in Interchromosomal Networks across the Cell Cycle

**DOI:** 10.1371/journal.pcbi.1003857

**Published:** 2014-10-02

**Authors:** Andrew J. Fritz, Branislav Stojkovic, Hu Ding, Jinhui Xu, Sambit Bhattacharya, Ronald Berezney

**Affiliations:** 1 Department of Biological Sciences, University at Buffalo, State University of New York, Buffalo, New York, United States of America; 2 Department of Computer Science and Engineering, University at Buffalo, State University of New York, Buffalo, New York, United States of America; 3 Department of Computer Sciences, Fayetteville State University, Fayetteville, North Carolina, United States of America; University of Tokyo, Japan

## Abstract

The interchromosomal organization of a subset of human chromosomes (#1, 4, 11, 12, 16, 17, and 18) was examined in G1 and S phase of human WI38 lung fibroblast and MCF10A breast epithelial cells. Radial positioning of the chromosome territories (CTs) was independent of gene density, but size dependent. While no changes in radial positioning during the cell cycle were detected, there were stage-specific differences between cell types. Each CT was in close proximity (interaction) with a similar number of other CT except the gene rich CT17 which had significantly more interactions. Furthermore, CT17 was a member of the highest pairwise CT combinations with multiple interactions. Major differences were detected in the pairwise interaction profiles of MCF10A versus WI38 including cell cycle alterations from G1 to S. These alterations in interaction profiles were subdivided into five types: overall increase, overall decrease, switching from 1 to ≥2 interactions, vice versa, or no change. A global data mining program termed the *chromatic median* determined the most probable overall association network for the entire subset of CT. This probabilistic interchromosomal network was nearly completely different between the two cell lines. It was also strikingly altered across the cell cycle in MCF10A, but only slightly in WI38. We conclude that CT undergo multiple and preferred interactions with other CT in the nucleus and form preferred -albeit probabilistic- interchromosomal networks. This network of interactions is altered across the cell cycle and between cell types. It is intriguing to consider the relationship of these alterations to the corresponding changes in the gene expression program across the cell cycle and in different cell types.

## Author Summary

During interphase, chromosomes occupy discrete bodies within the cell nucleus termed chromosome territories (CT). The organization of CT and the genes within them have been implicated in genomic regulation. Numerous reports suggest that specific interchromosomal interactions enable distally located genes to cluster in close proximity for coordinately regulated gene activity. Our overall understanding of CT organization and their interchromosomal interactions, however, is very limited. We have addressed this issue by combining the approaches of 3D FISH (fluorescence in situ hybridization), an extensive computational program that enables us to obtain precise measurements of CT interactions in 3-D and a novel global data mining program that evaluates the overall arrangement of all the interacting CT. 7 CT pairs ranging in size and gene density were examined in two different human cell types. Our findings demonstrate that: 1) there are preferred interchromosomal networks that are altered across the cell cycle; 2) these alterations are cell type specific; 3) all the CT interact with a similar number of other CT with the exception of the gene rich CT17 which interacts with significantly more CT; and 4) multiple interactions in the four possible combinations between CT pairs are far more common than in random simulations.

## Introduction

At the highest structural level, chromosomes form discrete bodies termed chromosome territories (CT) [1]–[6]. The three-dimensional organization of these CT and the genes within them have been implicated in genomic regulation [5]–[14]. Furthermore, interactions between CT and other nuclear domains have been shown to impact nuclear functioning [15]. For instance, chromosomes that contain the nucleolar organizing regions preferentially interact with nucleoli [16], [17]. While many studies have revealed a probabilistic nonrandom radial organization of CT within the nucleus [18] for review], fewer reports have investigated whether there are specific interchromosomal arrangements [19]–[21] and whether these are altered in different cell and tissue types [22]–[25] during cell differentiation and development [26] and in cancer [27]–[29]. The predominant method for studying CT organization has involved CT centers of gravity which may or may not reflect interactions of these CT at their borders. Also, due to technical limitations, the vast majority of these studies have been limited to three or less CT pairs per nucleus.

Numerous investigations have demonstrated that large-scale chromatin arrangements within the nucleus are relatively stable [30]–[35] and persist at least to some extent during the cell cycle stages of interphase [36], [37]. In contrast, analysis of in vivo labeled CT regions indicated that CT centers of gravity are mobile relative to the nucleus [38] and to each other [36]. While this is consistent with the prospect that the CT may undergo reorganization during the cell cycle, these investigations were not able to identify specific CT in their analyses [36], [38].

With this in mind we have analyzed the 3-D positioning and interchromosomal associations of 7 CT (1,4,11,12,16,17,18) using reFISH [24] in human WI38 lung fibroblasts and MCF10A (10A) breast epithelial cells in the G1 and S cell cycle stages. Differences in associations were found at both the cell type and cell cycle levels. Using a newly developed data mining algorithm termed the *chromatic median* [39], probabilistic network models were generated for the global patterns of interchromosomal interactions of all 7 chromosome pairs. The preferred arrangements were strikingly different between 10A and WI38 in both G1 and S phases.

Our findings provide support for a probabilistic “chromosome code” where the overall interactive network of CT is different between cell types and may contribute to the global regulation of gene expression [24], [25], [29]. We further report that this interaction network is altered across the cell cycle in a cell type specific manner.

## Results

**Figure 1:**
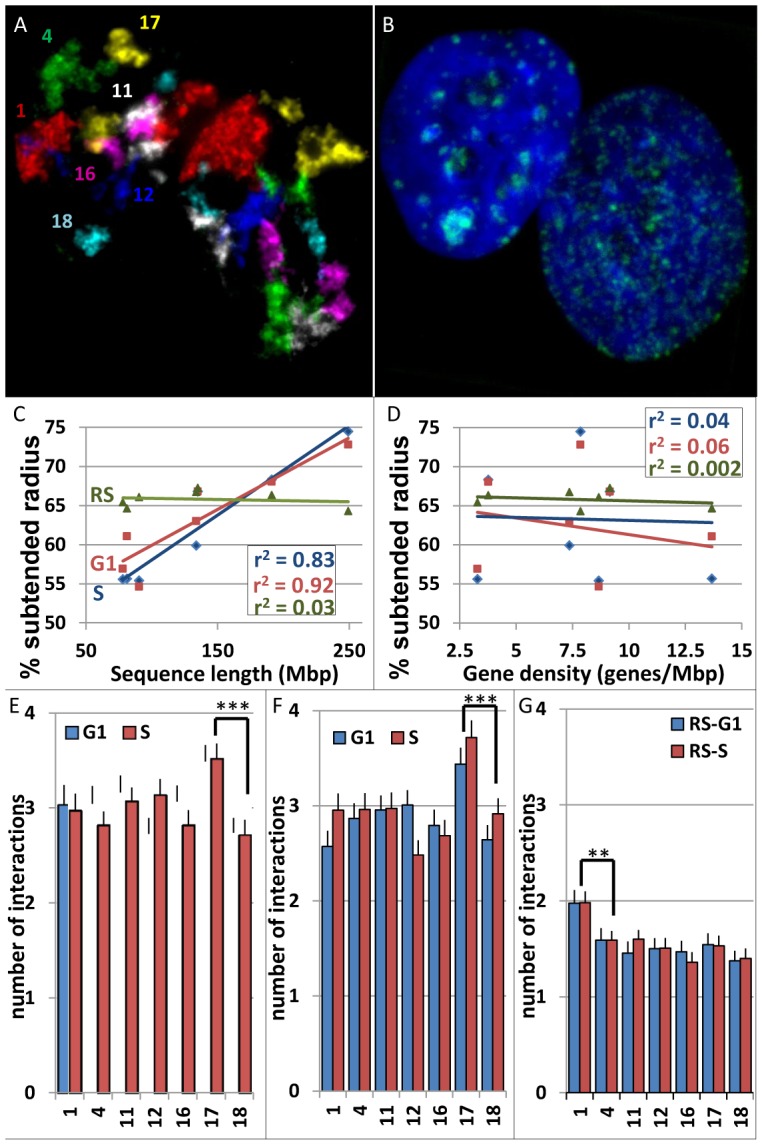
Multi-FISH labeling, radial positioning, and total interactions of CT during the cell cycle. 2-D projection images of: (**A**) 7 CT (1,4,11,12, 16,17,18) and (**B**) EdU labeled replication sites and DAPI staining in 10A cells. (**C–D**) Radial positioning- The percent subtended radii (the distance from the center of the nucleus to the CT center divided by the distance from the center of the nucleus through the CT center to the nuclear periphery in 10A cells) is displayed against chromosome sequence length (**C**) and gene density (**D**). Blue is G1, red is S, and green is random simulations. (**E–F**) Total pairwise CT interactions- The average number of CT within the subset of CT that each individual CT interacts with is shown for WI38 (**E**), 10A (**F**), and random simulations (**G**). Blue is G1 and red is S. All experimental values were significant higher than random simulations. CT17 interacts with a significantly greater number of CT than any other CT in the subset. CT1 is significantly higher than the other CT in random simulations. ***p<0.001.

In this study we combine the approaches of re-FISH, computer imaging, computational geometric and data mining to investigate chromosome territory interactions during the cell cycle (see Materials and Methods). A subset of 7 chromosomes (CT1, 4, 11, 12, 16, 17, and 18) were selected which are representative of the human genome by having a broad range in size and gene density. The weighted average gene density of this CT subset was 6.5 compared to 6.7 for the entire genome. In brief, WI38 human fibroblasts or MCF10A human epithelial breast cells were grown on gridded coverslips and labeled with EdU (labels S phase [40]), geminin (labels S and G2 [41]) and three rounds of chromosome paints. The G1 and S stages of the cell cycle for each cell type were then distinguished as described in Materials and Methods. Representative images for EdU and the CT are displayed in Figure 1A,B.

### CT volumes and radial chromosome positioning in WI38 and MCF10A cells

In both cell types, there was an average total nuclear volume increase of ∼10–15% in S phase compared to G1 and comparable increases in the CT volumes (Fig. S1). A correlation to chromosome size to radial position was demonstrated with the larger CT being more peripherally located (r^2^ = 0.72 to 0.92, Fig. 1C, Fig. S1), but no correlation to gene density (Fig. 1D, Fig. S1). Random simulations showed no correlation between chromosome size or gene density and radial position (Fig. 1C,D, Fig. S1). None of the radial positions were significantly altered from G1 to S phase in WI38, 10A, or random simulations. We did, however, determine significant cell type differences (t test, p<0.05) in radial positioning for CT1 in G1 and CT1, 11, and 17 in S phase.

### Pairwise CT interaction profiles are altered across the cell cycle

Although CT centers provide some insight into the positions of CT and organization of the genome (Fig. S2, S3), they do not accurately predict whether a CT pair will be interacting at their borders. We, therefore, determined the nearest border distances between CT as a direct measurement of the nearest distance in 3-D between all CT pairs. Using a maximum of 4 pixels (0.28 µ) as a threshold value for an interacting CT pair, we calculated the number of interactions each CT has to the other CT in the subset. An average of ∼90% of the measured distances that were determined as positive interactions (≤4 pixels) were actually separated by zero pixels and involved direct overlap (average of ∼15% of the volume) of the two interacting CT. Based on these measurements, each CT interacts on average with ∼3 out of the other 12 possible heterologs (Fig. 1E,F), with the gene rich CT17 displaying a significantly higher level of interactions (∼3.5–3.7 CT; t test, p<.001; Fig. 1E,F, Fig. S4). These levels of interaction are conserved in progression from G1 to S and are virtually identical in WI38 and 10A cells. In random simulations, that utilize the experimental CT volumes within the DAPI signal (see Materials and Methods), we find significantly lower levels (p<0.001) of interaction with an average of ∼1.5 interactions per CT (Fig. 1G).

Since a CT pair can interact up to four times within each nucleus (eg. CT1a-CT2a,1a-2b,1b-2a, and 1b-2b), we determined the percent of cells with 1 or more interactions (≥1), only 1 interaction ( = 1), or greater than 2 (≥2). If there was not a specific overall pattern to the CT associations in the cell nucleus, each pairwise interaction would be close to the average for all of the CT pairs. While the overall patterns for ≥1 (Fig. S5) were not significantly different from their averages in both WI38 and 10A cells, significant differences were found when single and multiple interactions were considered separately (chi squared, p<0.001, Table S1). Moreover, differences were found in comparing the single and multiple interaction profiles in the G1 and S phases of these two cell lines (p<0.002, Table S2). Corresponding random simulations do not show any significant differences across the cell cycle (Table S2).

**Figure 2:**
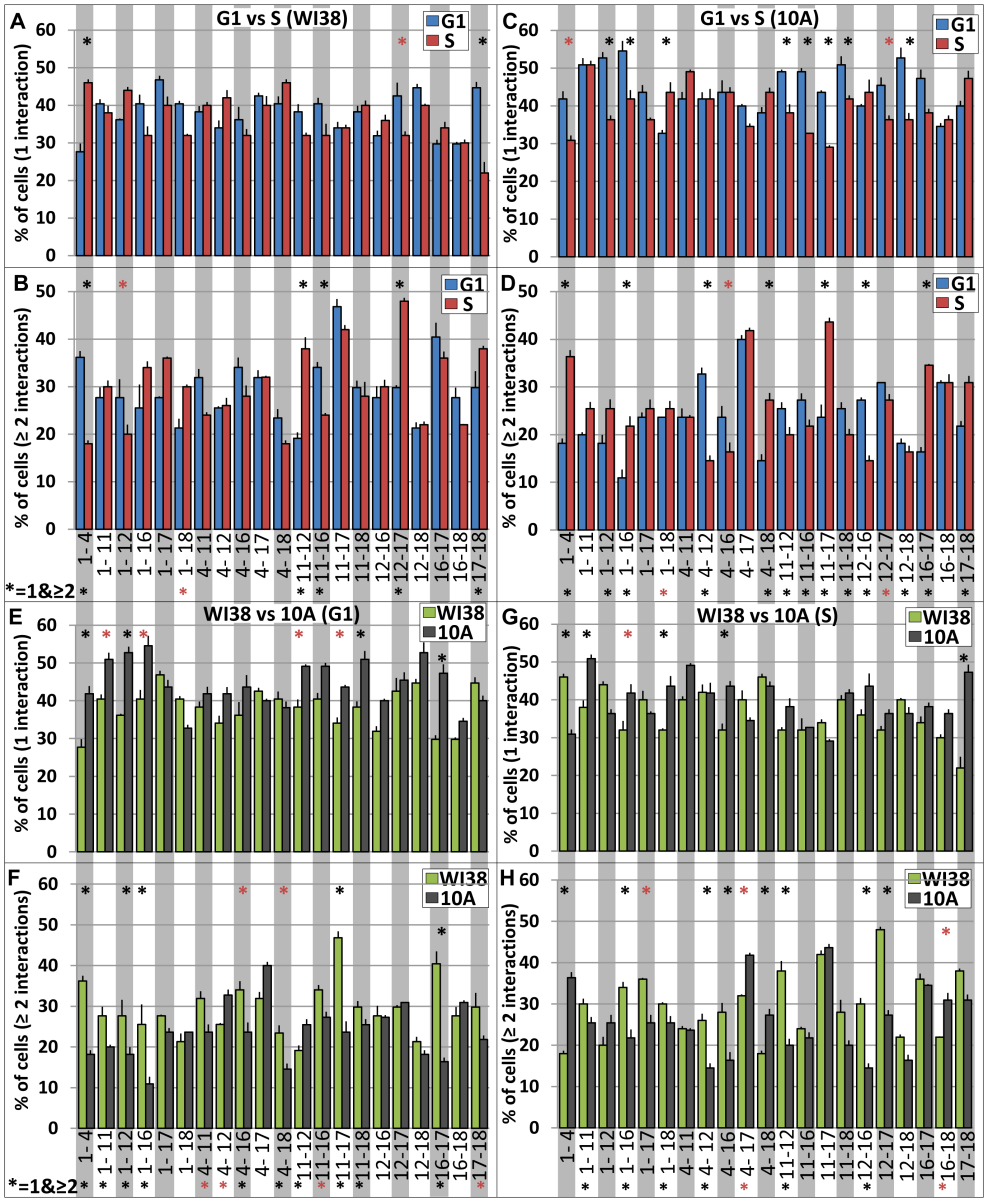
Interaction profiles of CT are altered across the cell cycle and cell types. Since each CT has two homologs there are four possible interactions in each nucleus. (**A–D**) Differences across the cell cycle- The percent of cells with only 1 interaction (**A**) and the percent of cells that have 2 or more interactions (**B**) in WI38 are shown for each of the 21 pairwise combinations of CT (G1, n = 46; S, n = 47). The percentage with only 1 (**C**) and 2 or more (**D**) in 10A are shown (G1, n = 56; S, n = 54); blue bars are G1 and red are S. (**E–H**) differences between cell types- The percent of cells with only 1 interaction (**E**) and the percent of cells that have 2 or more interactions (**F**) in G1 of WI38 and 10A are shown. The percent with only 1 (**G**) and 2 or more (**H**) in S of WI38 and 10A are shown, green bars are WI38 and black are 10A. error bars = SEM. Black asterisks indicate chi-square test, p<0.05, while red indicate p<0.10. Full p values are presented in Tables S3,S6.

**Figure 3:**
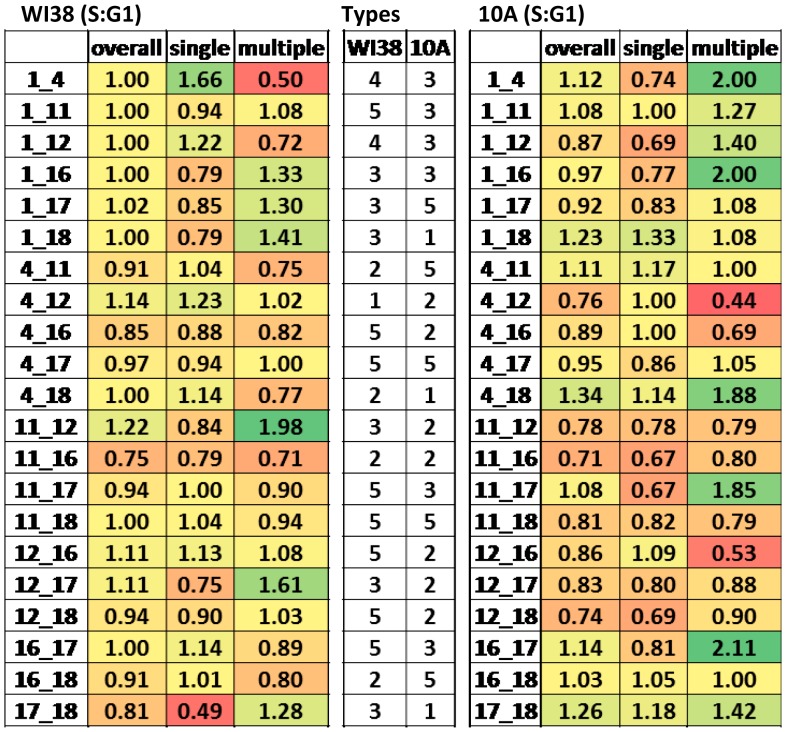
Alterations in interaction profiles from G1 to S phase. Calculation of the ratio between pairwise CT (S∶G1) reveals different alterations of CT interactions from G1 to S. Some alterations demonstrate an overall increase (type 1) in interaction while others have an overall decrease in interaction (type 2). Others switch from having greater levels with a singular interaction to those with more nuclei with multiple interactions (type 3) or vice versa (type 4). Others do not change from G1 to S (type 5). Values are color coded with green increasing the most from G1 to S and red decreasing the most. The types of alterations in interaction are displayed for WI38 and 10A in the center rows.

Of the 21 pairwise CT combinations in the ≥1 interaction profiles of G1 versus S phase, only 2 were altered (based on a >20% difference) in WI38 and 7 in 10A (Fig. S5). For cells with only 1 interaction, 8 of the 21 pairwise CT combinations were altered from G1 to S phase in both WI38 and 10A (Fig. 2, Fig. 3) while 11 (WI38) and 13 (10A) of 21 were altered for multiple interactions (≥2). When considering both = 1 and ≥2 together, we found 5 pairwise CT interaction profiles that were altered from G1 to S phase in Wi38 (Fig. 2B, Table S3), and 13 in 10A (chi-square, p<0.05, Fig. 2D. Table S3). Corresponding random simulations showed no significant differences (Fig. S6, S7). While the experimental levels of interaction did not show any relation to CT size, larger CT had higher amounts of interaction in random simulations and ranged from a low of 32% for CT12–18 to 56% for C1–17 (Fig. S6, S7). Homologous interactions were lower than heterologous ones even when correcting for the fact that there is only one possible homologous interaction (Table S4). In addition, experimental homologous interactions are similar in range to random simulations and do not change significantly across the cell cycle.

Multiple interactions are typically less abundant than singular interactions. 16–60% of cells that contain an interaction, however, had multiple interactions (Fig. 2). This is far greater than random simulations (7–28%, Fig. S6, S7). CT17 was represented in 83% of the CT pairs that were in the top half of multiple interactions compared to 42% for CT18. The highest multiple interacting pair in WI38 was CT11–17 in G1 and CT12–17 in S (Fig. 2B) and CT4–17 (G1) and CT11–17 (S) in 10A cells (Fig. 2D).

**Figure 4:**
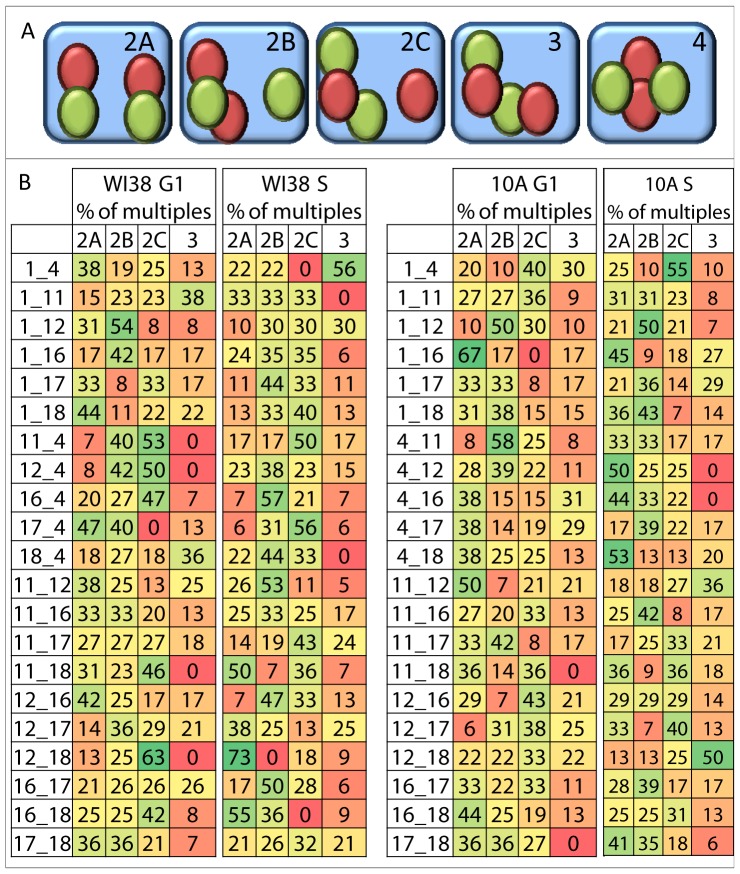
Patterns of multiple interactions of CT pairs. (**A**) Categories of multiple interactions of CT pairs for 2, 3 and 4 interactions are illustrated; (**B**) the percent of multiple interactions that are type 2a, 2b, 3 interactions were calculated and are shown for WI38 and 10A. Green depicts the highest levels of interaction between CT pairs and red the lowest. Chi-square test indicates many significant differences between 2A–2C between CT pairs across the cell cycle and between cell types.

We found three distinct patterns when a CT pair interacts twice (termed 2a, 2b, and 2c, Fig. 4A). In pattern 2a, one homolog of each CT is a member of an independent pairwise interaction with its heterologous partner CT. In pattern 2b, a triplet is formed involving one copy of one CT and 2 copies of the other heterologous CT (Fig. 4A). In pattern 2c, the opposite triplet is formed (Fig. 4A). Out of the multiple combinations the percent of cells with all four CT forming an alternating chain with 3 interacting CT (pattern 3, Fig. 4A) were typically less abundant than the other multiple patterns but was the most abundant pattern in 5 out of the 84 pairwise combinations studied (Fig. 4B). Four interactions were rare (∼0–5%) as they only occurred when all four homologs of the two chromosomes were in close apposition. Differences were found between G1 and S in the overall patterns of these configurations for CT pairs (Fig. 4B). For example, some pairwise combinations of CT are equally distributed between 2A–2C patterns (e.g., CT1–11 in WI38 S), while others are present predominantly in one configuration (e.g., CT12–18 in WI38 S). The patterns of these multiple interactions are also different between 10A and WI38 (Fig. 4B).

Further analysis revealed dynamic alterations in interaction profiles during the cell cycle (Fig. 3). Some CT pairs had an overall increase in both single ( = 1) and multiple (≥2) interactions (type 1), while others decreased in both = 1 and ≥2 from G1 to S phase (type 2). Still others switched from single to multiple interactions (type 3) or vice versa (type 4). Moreover, a significant proportion of the CT pairs did not change in interaction from G1 to S (type 5). In 10A, CT18 is a member of all three CT pairs that increased in overall interaction from G1 to S and CT12 is a member of 5 of the 7 CT pairs that decrease overall in interaction. CT1 switched to more cells with multiple interactions in 4 of the 6 possible pairwise interactions in 10A and 3 of 6 in WI38. These observed altered chromosome interactions are represented within the overall model of CT interactions (see chromatic median section).

### Cell type differences in interaction profiles across the cell cycle

While the overall interaction profiles of 10A to WI38 in either G1 or S phase were not significantly different for the percent of cells with one or more interactions (Fig. S5, Table S5), significant differences (chi squared, p<0.001, Table S5) were found for the corresponding single and multiple interaction profiles (Fig. 2 E–H, Table S5) and when considering singles and multiples together. In comparing the 10A and WI38 single interaction profiles, 12 (G1) and 9 (S) of the 21 pairwise CT combinations were different by >20% (Fig. 2 E,G). For the multiple interaction profiles, 13 (G1) and 14 (S) of the 21 CT combinations were different by >20% (Fig. 2F,H). When considering both single and multiple interaction profiles together, 10 pairwise CT interactions were significantly different in G1 (Fig. 2E,F, Table S6, chi-square, p<0.05,), and 11 were significantly different in S (Fig. 2G,H, Table S6, chi-square, p<0.05). Interestingly, the interaction profiles in each cell type changed in a different way across the cell cycle. For example CT1–4 was a member of type 3 in WI38, but was switched to the opposite (type 4) in 10A (Fig. 3). Simulations displayed no significant differences between 10A and WI38.

### The preferred probabilistic model of CT interactions is altered across the cell cycle and between cell types

**Figure 5:**
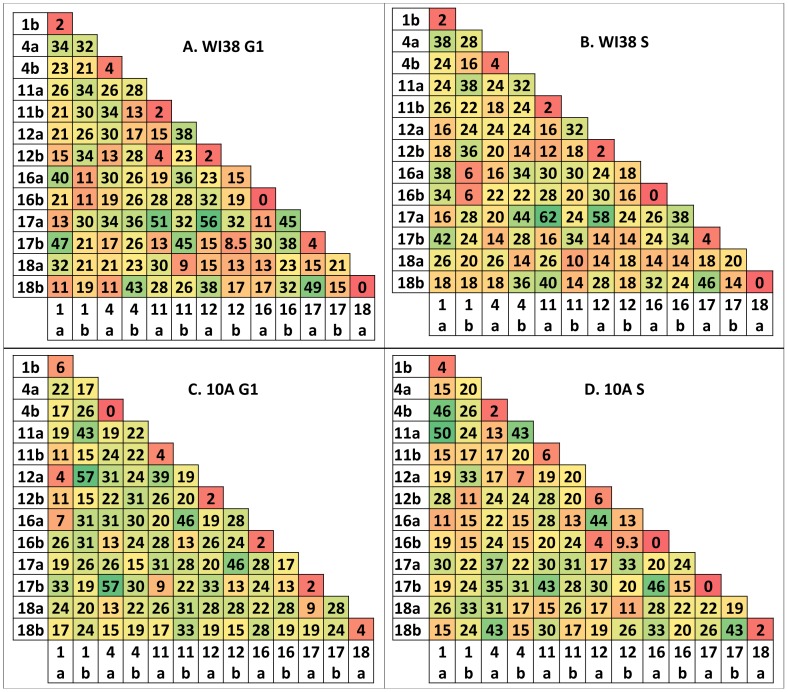
Chromatic median analysis of chromosome interactions in the cell cycle of WI38 and 10A. The chromatic median algorithm determines correspondence between homologs across nuclei based upon their interactions with other CT. This algorithm determined a median matrix for CT interactions in WI38 (**A–B**) and 10A (**C–D**) in G1 (**A, C**) and S phase (**B,D**). Each cell in the matrix represents the percent of input nuclei that have an interaction between those homologs. The values are color coded on a colorscale from low (red) to high (green).

The difference in the profiles of CT interactions between G1 and S phase of WI38 and 10A cells suggests reorganization in interchromosomal interactions during the cell cycle. Furthermore, it reinforces the concept of cell type specificity to CT organization beyond the Go state. To investigate this further we used an algorithm designed to determine the overall pattern of CT interactions across the population of cells (see Materials and Methods, Fig. S8). This program, termed the chromatic median, represents each nucleus as a 14×14 (7 CT for this study, 2 homologs per CT) binary matrix wherein a value of 1 indicates an interaction and a value of 0 indicates the absence of an interaction. It then determines the corresponding homologs across all nuclei based upon the homologs' interactions with all the other individual chromosome heterologs under investigation, thus switching the ‘a’ and ‘b’ labels for homologs based on its interactions with other CT. Subsequently, we determine the percent of cells that contain an interaction for each of the 91 positions in the matrices (Fig. 5). Within the resulting median matrices, we found hot and cold spots that range from 0 to 62% of input cells (Fig. 5). Random simulations did not show hot spots and the range of values was approximately 2.3 fold less than experimental values (0–27%, Fig. S9).

**Figure 6:**
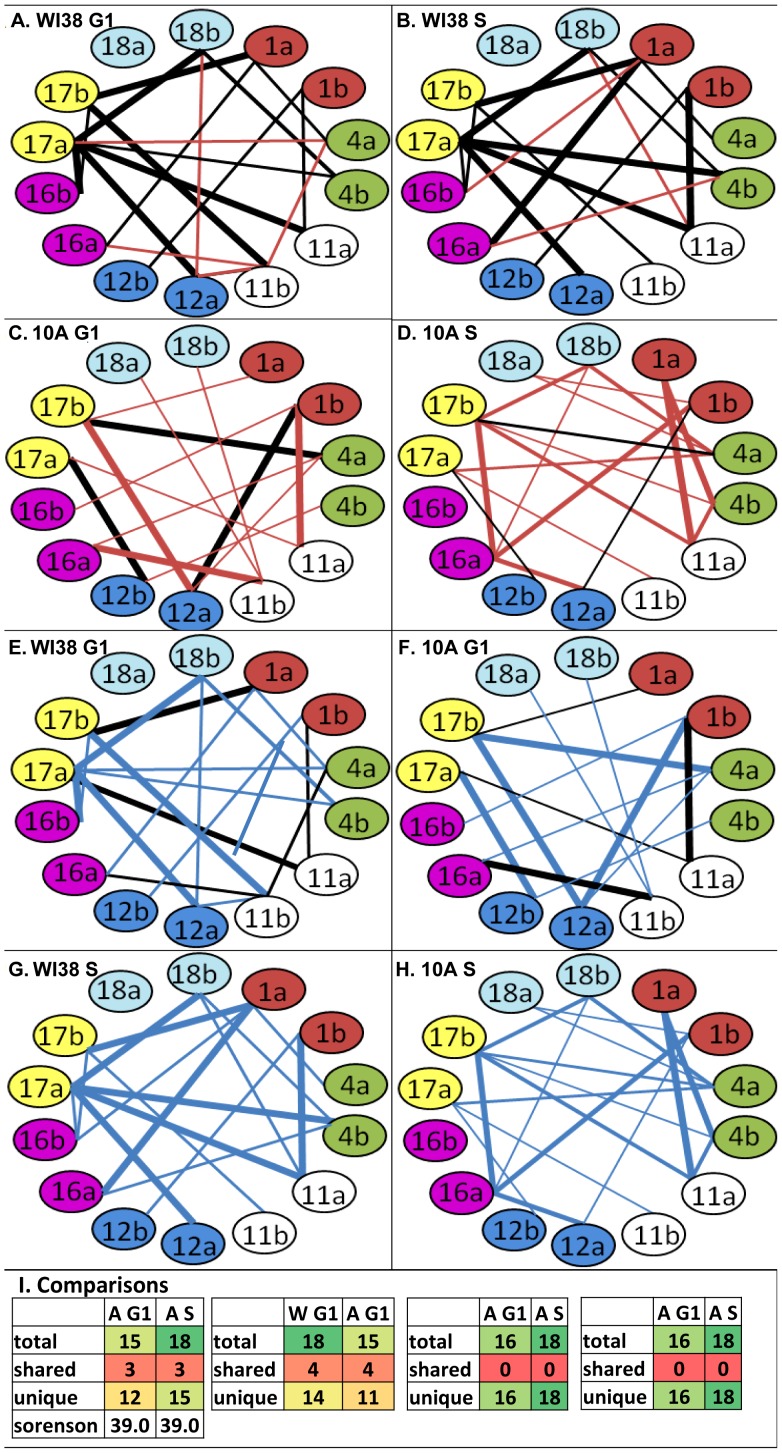
Preferred probabilistic network models of chromosome interactions in the cell cycle of WI38 and 10A. Thresholding of the WI38 (32%) and 10A (31%) matrices above a level where there are no connections in random simulations or randomizations of input matrices or random simulations level interactions (Fig. S9) reveals 14–18 CT interactions in G1 of WI38 (**A, E**), S of WI38 (**B, F**), **G1 of** 10A (**C, G**), and S of 10A (**D, H**). Red lines are unique connections in G1 or S phase (**A–D**), blue lines indicate unique connections between cell types in each stage of the cell cycle (**E–H**) and black lines represent common connections. Comparison of the models generated from this analysis indicate more differences between G1 and S phases in 10A compared to WI38 cells and virtually completely different networks between cell types across the cell cycle (**I**). Thick connections are within the top third of percent pairwise interactions within the model. Connections of medium thickness are in the middle third and thin lines are within the bottom third. Sorensons analysis [42] determined that a given nucleus will contain an average 39–42% of the connections within these models.

To determine a preferred probabilistic model of CT interactions, thresholding was performed on the matrices at 32 (WI38) and 31% (10A) association. This enriched for the CT interactions found at the higher levels among the total population. Moreover, at these thresholds, there are no connections in randomizations of input matrices or random simulations (Fig. S9). 15–18 CT interactive connections were identified in both the 10A and WI38 (Fig. 6). Comparison of the models generated from this analysis revealed only modest differences between G1 and S phase of WI38 cells, but massive differences across the cell cycle of 10A. 13 connections were shared between G1 and S of WI38, but only 3 were shared between G1 and S of 10A. The differences between cell types were even more striking. Only 4 were shared in G1 and none were shared in S phase. Thick connecting lines are within the top one third of associations within the model Fig. 6). Sorenson's analysis [42] revealed that each individual nucleus contains on average 39–42% of the connections displayed in these probabilistic models (Fig. 6).

Since random simulations had much lower levels of interaction than experimental (Fig. S9), we evaluated whether the high level of interactions in the experimental data falsely lead to a model of interactions instead of random interactions. After running the chromatic median on randomized matrices, the range of values (13–32%, Fig. S9) was approximately 3.3 fold lower than experimental values which range from 0–62% (Fig. 5). The range for experimental is 62 and the range for randomization was 19. 62 divided by 19 is 3.26. This demonstrates that the process of determining corresponding homologs in a cell population with a high degree of interactions does not artificially create a pattern of those interactions.

## Discussion

It is well established that histones are dynamically regulated through a diverse array of modifications [43], [44], which can result in visual alterations in chromatin structure [45], [46]. Chromatin remodeling has been shown to control progression through the cell cycle by regulating transcription of essential cell cycle genes [47], [48] and replication. For example, during S phase loosening of chromatin structure enables the replication machinery to access the DNA [49]. At a global level, as cells progress from early S to mitosis, the chromatin becomes increasingly condensed and less sensitive to DNase I [50].

Chromatin organization is also thought to be involved in the compartmentalization of the genome and long range interaction between genes [51]. These interactions have been shown to occur in a transcription dependent manner to regulate gene expression [52]–[55]. Fundamental to these interactions is the arrangement of chromatin into discrete chromosome territories (CTs) in the interphase nucleus (for review see, [18]). Despite this progress, our understanding of the spatial positioning of the CTs, their interchromosomal associations and the resulting influence on gene regulation is less clear [7]–[14], [56]–[58]. For example, only a few reports have investigated whether there are preferred interchromosomal positional arrangements between CT [19]–[29]. However, the majority of these studies were primarily focused on the gravity centers of CT which may not reflect their interactions at their interfaces and/or were limited to 3 chromosomes per nucleus.

Earlier reports suggested a high degree of stability of the large scale arrangement of chromatin in the cell nucleus [30]–[35] and during the cell cycle [36], [37]. If specific interchromosomal interactions are involved in gene expression, however, alterations in these interactions should occur during the cell cycle in coordination with the known changes in gene expression [59]–[62]. Detection of positional changes during the cell cycle of in vivo labeled CT regions is consistent with this possibility [36], [38]. However, the identity of the CT could not be determined in these previous studies.

To directly address whether specific CT interactions are altered across the cell cycle, we examined the CT spatial positioning and interchromosomal associations using reFISH [24] to concurrently label 7 CT (1,4,11,12,16,17,18) in the G1 and S phases of WI38 lung fibroblast and MCF10A human breast epithelium cells. An integrated suite of in-house developed software [24], [63] and a new 3D distance measurement program termed *eFISHent* were then applied to the collected images to generate an extensive database of distance measurements. A new data mining and pattern recognition algorithm termed the chromatic median [39] was then applied to determine whether there is an overall preferential organization of the CT interactions.

### Radial positioning of chromosome territories

The radial positioning of genes has been suggested to be functionally linked to gene expression. In support of this proposal, highly expressed genes generally are found more internal than inactive genes [64]–[70] and heterochromatin is found preferentially at the nuclear periphery [71], [72]. The major contributing factors involved in peripheral positioning of CT are posited to be size [20], [24], [25], [29], [73], gene density [74], [75], or both these properties [76]. Differences in the nuclear shape of cells may also play a role in CT radial positioning [20], [77].

While it has been reported that CT change their radial organization when cells re-enter the cell cycle from quiescence [78], [79], our studies demonstrate that CT do not change their radial positioning during the cell cycle. In contrast, we found that the radial positioning of some CT are different between cell types. These differences between cell types are stage specific. For example CT11 and CT17 are not significantly different in their radial positioning comparing WI38 and 10A in G1, but are in S phase. Importantly, random simulations demonstrated that differences in nuclear shape or CT volume do not account for these changes in radial positioning.

### Alterations of interchromosomal organization in the cell cycle

Previous studies of interchromosomal associations based on measurements of the pairwise border distances between individual CT, have suggested an overall non-random nature of these interactions [19]–[29]. Our analysis of interchromosomal associations has revealed for the first time, multiple CT associations with an average of ∼3 interactions out of a maximum of 12 for each CT copy or 20–30% of the other heterologs in the CT subset. This extrapolates to 9–13 interactions per chromosome out of the 44 possible heterologous interactions at the whole genome level. Thus each CT in the nucleus is interacting with an amazingly high proportion of the other CTs. There was no relationship between chromosome size and total levels of interaction. For example, even though chromosome 1 is approximately 3.2 fold larger in sequence length than chromosome 18, both have similar levels of interaction. The radial positioning of CT may allow for this equivalent level of interaction as larger CT have large borders with the nuclear periphery. In this view, smaller CT have a larger proportion of their surface area free for interaction with other CT than larger CT. Moreover, the gene rich CT17 interacts with significantly more CT than the gene poor CT18 or CT1. This suggests a possible relationship of the level of interchromosomal associations for a CT to its gene density and/or activity.

While CT17 interacts with more CT in experimental nuclei, in random simulations that account for nuclear and CT volumes, as well as general irregularity in CT shapes, we do not find a gene density correlation to interaction. In a separate investigation from our group we have reported a large variation in chromosome shapes that are related to gene density. Thus gene-rich chromosomes are much more irregular in shape than gene poor ones (Sehgal et al, 2014, manuscript under review). This investigation found that CT17, for example, was much more irregular in shape than the gene poor CT18 in WI38 cells (Sehgal et al., 2014 manuscript under review) Such irregularity could lead to the greater level of interactions displayed by CT17. In this model, extensions from gene rich CT could allow for greater levels of interactions with other CT. It is further proposed that these interchromosomal interactions are critical for the coordinated regulation of the genome. Determining the intrachromosomal organization of CT17 during the cell cycle would enable identification of regions which are frequently external to CT17 and may, therefore interact with regions within other CT.

While the total level of CT interactions did not change from G1 to S, there were significant alterations in singular and multiple CT pairwise interactions. Five types of alterations were determined from G1 to S. Some CT pairs increased in both singular and multiple interactions (type 1), while others decreased in both (type 2). Other CT pairs increased in singular interactions while decreasing in multiples (type 3) or vice versa (type 4). Finally, some CT pairs did not change in their level of interactions (type 5). Moreover, cell type differences were detected for the cell cycle alterations. For example CT1–4 are type 3 in WI38, but type 4 in 10A. Only 4 of the 21 pairwise interactions are altered similarly in WI38 and 10A (eg. CT1–16, Fig. 3).

Previous reports have demonstrated cell type specificity to chromosome interactions in G0 cells [24], [25]. We, therefore, applied a novel computational data mining and pattern recognition approach termed the *chromatic median* to identify overall patterns of global interactions across the cell cycle in both cell types. A maximum of 18 out of 91 possible connections were found to be above random simulations and randomizations of the input population. While 13 connections within the model of WI38 in G1 are shared in S, only 3 are shared in 10A from G1 to S (Fig. 6). Between cell types, only 4 were shared in G1 and none were shared in S indicating cell type differences across the cell cycle. This suggests that certain cell types may demonstrate more alterations in interchromosomal organization across the cell cycle than others.

Our findings support the presence of a higher order probabilistic chromosome code or network of CT interactions inside the cell nucleus [24], [25], [29]. It is proposed that the preferred interchromosomal association network defined by this code is maintained epigenetically and facilitates specific genomic expression programs characteristic of the particular cell type such as the fibroblast and epithelial cells of this study. The probabilistic nature of the overall interactive network could in turn provide flexibility for alterations in the network and contribute to corresponding changes in the overall genomic program. Consistent with this view, the interchromosomal networks are dynamically altered during the cell in conjunction with gene expression changes [59]–[62].

While many studies using microscopy have demonstrated simplistic and distinct chromosome clusters of interaction involving a limited number of interfaces between CT [5], [6], [10], [14], [18], our studies have demonstrated a complex network of interactions with a high degree of interchromosomal interactions [24], [25], [29]. In comparison, studies using chromatin capture techniques, such as genome-wide Hi-C, have also demonstrated a complexity to interchromosomal interactions. Initially these studies were performed as a population average and demonstrated interactions between all possible pairs of chromosomes [80]–[84]. More recently using single cell Hi-C, Nagano et al, 2013 discovered cell-specific clusters between domains on different chromosomes [85]. These domains were found to be of similar level of activity such that active domains preferentially paired with active (and inactive domains with inactive). Notably, the authors identify multiple domain–domain contacts between CT interfaces. While single-cell Hi-C also demonstrates cell-to-cell variability, our findings indicate that particular homologs of CT display patterns of interaction across the population of cells (Fig. 5, 6). Within a pair of CT, we further define the pairwise interaction profile wherein certain CT pairs can demonstrate a greater degree of multiple interactions than others (Fig. 2, 3). This suggests that while some of these multiple interacting domains between CT interfaces are possibly on the same interacting homologs, they may be distributed between the four possible pairwise interactions (especially on CT pairs that interact as multiples more frequently). In direct agreement with our findings, this investigation similarly found a high level of interchromosomal interactions and that the total level of interactions between chromosomes was independent of chromosome sequence length [85].

Using a on synchronized HeLa cells, it was demonstrated on a population level that the intrachromosomal topologically associating domains identified by this approach are largely maintained across the cell cycle [86]. Further studies using Hi-C approach for studying interchromosomal interactions at the genomic level in both cell populations and at the single cell level [80], [85], should enable more detailed definition of the many alterations in chromosomal interactions identified in our investigation. In particular, differences between cells in different stages of the cell cycle within a population could be distinguished via cell sorting followed by single cell Hi-C [85].

## Materials and Methods

### Cell culture

MCF10A cells (Barbara Ann Karamanos Cancer Institute, Detroit, MI) were grown in DMEM/F-10 media supplemented with 5% horse serum, 2% insulin, EGF, hydrocortisone, cholera enterotoxin, and 1% penicillin/streptomycin. WI38 was cultured in DEME/F-10 media with 5% FBS and 1% penicillin/streptomycin. All cell lines were grown at 37°C in a 5% CO_2_ incubator.

### 3D microscopy and reFISH image analysis

Seven chromosome territory pairs were analyzed by sequential rounds of chromosome territory FISH labeling, image collection, stripping and re-FISH as previously described [24]. In the final round EdU replication labeling [40] was used to discriminate between cells that are in S phase versus those that were not. Geminin which labels cells in S/G2/M [41] was used to exclude cells that are in G2. Those cells which are geminin+/EdU- were not analyzed. Images were collected on an Olympus BX51 fluorescence microscope equipped with a Sensicam QE (Cooke Corporation, Romulus, MI) digital CCD camera, motorized z-axis controller (Prior, Rockland, MA) and Slidebook 4.0 software (Intelligent Imaging Innovations, Denver, CO). Three dimensional z stacks (0.5 µM intervals) of three or four chromosome territories per in situ hybridization were collected and deconvolved with a NoNeighbor algorithm in Slidebook 4. Images from each round of labeling were aligned by comparing x,y,z coordinates of landmark refractile structures in corresponding phase contrast using registration software developed in our laboratory [24], [63] and with imageJ's translation function. This process allows two different sets of images of the same nucleus to be combined into a single image set. Accuracy of matching was then verified by merging of DAPI images from different rounds followed by imageJ's line profile tool.

The chromosome territories were segmented into binary images using ImageJ's threshold feature. Three different criteria were used to distinguish between signal and noise including: (1) algorithms that process the intensity histograms using ImageJ threshold reference isodata; (2) user selection of a narrow range of intensities corresponding to the chromosomes borders; (3) decreasing the threshold until the captured pixels are no longer connected to the chromosome. All three of these criteria result in nearly identical selection of chromosome signals into binary segmented images. Next we developed a program termed *eFISHent* that reconstructs the 3D shapes of CT based on well-known region labeling algorithms to determine the boundary of each CT [87] and subsequently measures many parameters within the nucleus. The eFISHent program then measures in 3-D a large number of parameters including: their volumes, volume overlap between interacting CT, minimal border-to-border distances (pairwise border distances, *PBDs*), distances between centers of gravity (pairwise center distances, *PCDs*), distances between peripheries and centers (*PBCDs*), the distance of the line projecting from the nuclear center through the center of the chromosome/gene to the nuclear periphery (subtended radii, *SR*), minimal peripheral distance to the nuclear periphery (*MPD*), centroid xyz coordinates, and major and minor axes. This program is versatile since it will measure all of these values for any given amount of input objects simultaneously. For example, with 7 chromosomes labeled, as in this study, it will measure the 18 homologs' volumes, the nuclear volume, 252 pairwise heterologous distances (82 PBDs, 82 PCDs, and 82 PBCDs), 21 homologous distances (7 PBDs, 7 PCDs, and 7 PBCDs), 14 subtended radii, 14 MPDs, 14 centroid coordinates, and 28 major/minor axes. For validation we simulated data of known distances and found that our program accurately measures all distance combinations. We also used conventional measurement techniques in imageJ to validate distance measurements made by eFISHent in experimental FISH between BAC probe labeling.

Since the volume determination of each homolog in a CT pair are never exactly the same, this program enables us to distinguish “homolog a” as having a larger volume than “homolog b”. This results in four pairwise distances for each CT (e.g., 1a-2a, 1a-2b, 1b-2a, 1b-2b). Any given nucleus, therefore, will have between 0 to 4 associations for each CT pair. From this data, the percentages of pair-wise associations based on PBD measurements were calculated using a threshold distance of ≤4 pixels or ≤0.28 µM as the minimal nearest 3-D distance for a “positive interaction” [24]. ∼90% of these values for each chromosome pair were “zero” pixel values. We have found that all zero pixel values represent a degree of overlap or co-localization between the two CT under measurement [29]. Thus the 4 pixel threshold used in these studies for nearest neighbor CT pairs is indicative of interchromosomal interactions and not simply the close proximity of CTs.

### Random simulation of nuclei and chromosome territories

While many simulations are done using an artificial nucleus and preset volumes run many times [24], to more accurately mimic the experimental conditions, we have simulated the precise nuclear and CT volumes and shapes for each image set. All images from each given nucleus are contained within its own separate folder. The simulation program reads the volumes of the CT within each CT image, selects a point at random from within the DAPI mask and grows asymmetrically from that point until the volume and shapes similar to the experimental CT in that given nucleus are reached. If a CT reaches the nuclear border it no longer grows in that direction - ensuring that all simulated CT are within the nucleus.

### Chromatic median analysis and modeling chromosome territory associations

Previously an algorithm called the generalized median graph (GMG) was developed to determine the probabilistic best fit model for global interactions of chromosome in the Go stage of WI38 human fibroblasts [24], [25], [88]. The GMG considered all possible association matrices (i.e., all permutations of the association graphs) and simultaneously optimizes the associations of all CT under consideration. To tackle a larger population of cells with more CT and enhance the theoretical guarantee of the quality of the solution, we have developed a new algorithmic technique termed the *chromatic median* or CM which uses combinatorial optimization to infer the common chromosome interaction pattern or network for the overall cell population [39]. While the GMG used integer linear programming and rounding techniques, the CM is more accurate and robust. It is based on a number of new techniques, such as semi-definite programming, multi-level rounding, geometric peeling, and adaptive sampling [89], [90]. The CM technique results in much better approximation ratios and yields near optimal solutions in all tested random or real datasets [39].

Details of the CM technique and its mathematical basis are presented elsewhere [39]. In brief, this approach represents each nucleus as an 14×14 (7 CT for this study, 2 homologs per CT) binary matrix wherein a value of 1 designates an interaction and a value of 0 indicates the lack of an interaction. This is illustrated in Figure S8. The objective is to find the best permutation (re-categorizing from “a” to “b” and vice versa for all CT pairs within each nucleus) which will align the matrix of each input cell with that of the common pattern. The new CM algorithm considers all possible permutations of the interactions and simultaneously optimizes the interactions of all pairs of heterologs and homologs. For example, if there is a high frequency of nuclei wherein one homolog of CT4 associates with CT12, 16, 18, while the other homolog associates with CT1, 11, and 17, it will classify the first as CT1a and the second as CT1b across all cells. This process is done simultaneously for all CT studied to maximize similarity across the population. The number of input cells which have an interaction is then determined for each of the possible pairwise combinations.

After permutation analysis, the CM gives an output matrix which lists the percent of cells that have that given interaction. Using excel's conditional formatting, each interaction is filled with a color ranging from green (high/hot spots) to red (low/cold spots). Yellow indicates moderate values. After setting a threshold for interactions, probabilistic models are generated of preferred CT interactions among the entire subset of CTs. A simple example of this process is illustrated in Figure S8.

## Supporting Information

Figure S1 **Nuclear volumes and radial positions of CT in the cell cycle.** (**A**) The percent difference in CT absolute volumes from G1 to S are shown. The volume differences as a percent of the nucleus are also shown. (**B**) The relationship between radial positioning (% subtended radii) and sequence length (**B**) or gene density (**C**) in WI38 is shown. Blue is G1, Red is S, and green are random simulations.
(TIF)

Figure S2 **Comparison of experimental pairwise center distances (PCD) between all combinations.** Experimental pairwise distances between CT centers were normalized to the major axis. Each position within the matrix represents a comparison between the pairwise CT centers in 10A G1 (**A**), 10A S (**B**), WI38 G1 (**C**), and WI38 S (**D**). Green squares represent p<0.05 and red p<0.001.
(TIF)

Figure S3 **Comparison of pairwise center distances (PCD) between all combinations in random simulations.** Each position within the matrix represents a ttest comparison between the normalized pairwise CT centers in random simulations of 10A G1 (**A**), 10A S (**B**), WI38 G1 (**C**), and WI38 S (**D**). Green squares represent p<0.05.
(TIF)

Figure S4 **Distributions of total interactions between CT.** The number of interactions between each CT pair and other CT studied was determined. The distributions of interactions (≥1, ≥2, etc.) for each CT are shown for 10A G1 (**A**), 10A S (**B**), WI38 G1 (**C**), and WI38 S (**D**), and random simulations of 10A G1 (**E**) or 10A S (**F**).
(TIF)

Figure S5 **Interaction profiles of CT (≥1 interaction).** The percent of cells with at least one interaction in WI38 (**A**) and 10A (**B**) are shown. Blue bars are G1 and red S. Chi square p values are shown for the difference between G1 and S (**C**).
(TIF)

Figure S6 **Interaction profile of CT in random simulations in G1 and S phase of WI38.** Simulations were performed where CT of the same volume were grown asymmetrically simulating CT morphology inside the experimental nuclei (see materials and methods). The percent of cells with at least one interaction (**A**), only 1 interaction (**B**) and greater than 2 interactions (**C**) are shown. Blue bars are random simulations of G1 and red are random simulations of S. Error bars are SEM.
(TIF)

Figure S7 **Interaction profile of CT in Random simulations in G1 and S phase of 10A.** The percent of cells with at least one interaction (**A**), only 1 interaction (**B**) and greater than 2 interactions (**C**) are shown for random simulations using 10A G1 or S cells. Blue bars are random simulations of G1 and red are random simulations of S. Error bars are SEM.
(TIF)

Figure S8 **Schematic diagram of chromatic median analysis.** The interactions between CT in each input nucleus are represented as a binary code in input matrices. The input into the chromatic median program is defined based upon CT volumes with the larger CT homolog termed “a” and the smaller “b” (**A**). After a permutation analysis that defines homolog a versus homolog b based upon each homologs interaction with other CT (**B**), the percent of cells with an interaction between all pairwise combinations is determined (**C**). CT homologs that are switched are indicated by double arrows and are outlined in bold in their corresponding matrices. The values are color coded on a color-scale from low (red) to high (green).
(TIF)

Figure S9 **Chromatic median analysis of random simulations and randomizations in G1and S.** The chromatic median algorithm determines correspondence between homologs across nuclei based upon which other CT it interacts. This algorithm determined a median matrix for CT interactions for random simulations that put CT of similar volume within the DAPI signal in WI38 (**A–B**) and 10A (**C–D**); in G1 (**A, C**) and S phase (**B,D**). Next we randomized the input matrices of the experimental input cells for WI38 G1 (**E**), WI38 S (**F**), 10A G1 (**G**), and 10A S (**H**). Each cell in the matrix represents the percent of input nuclei that have an interaction between those homologs. Values are color-coded on a color-scale from low (red) to high (green).
(TIF)

Table S1 **Chi-square values comparing the overall patterns to uniform averages.** The overall patterns were compared to their uniform averages. Chi-square p values are shown for experimental and random simulations. Green p<0.05, yellow p<0.01, red p<0.001.
(DOCX)

Table S2 **Chi-square values comparing the overall patterns between G1 and S.** The chi-square p values are shown comparing the overall patterns in G1 to S in 10A to WI38 and in random simulations for G1 versus S. Green p<0.05, yellow p<0.01, red p<0.001.
(DOCX)

Table S3 **Chi-square values of individual CT pairs across the cell cycle.** The chi-square p values are shown comparing G1 to S for each individual CT pair in WI38 and in 10A for differences in the percent of cells with only 1 interaction, ≥2 interactions, and when considering both = 1≥2 interactions together. Purple p<0.10, Green p<0.05, yellow p<0.01, red p<0.001.
(DOCX)

Table S4 **Homologous versus heterologous Interactions.** The percent of total interactions for heterologous (4 possible per cell) and homologous (1 possible per cell) CT interactions were ranked from lowest to highest in G1 of WI38 or 10A. Heterologous interactions are normalized for the fact that there are 4 possible interactions. Homologous CT interactions are written in magenta. The values are color coded on a color-scale from low (red) to high (green).
(DOCX)

Table S5 **Chi-square values comparing the overall patterns between cell types within G1 or S.** The chi-square p values are shown comparing the overall patterns in WI38 to 10A in G1 and in S and comparing random simulations of WI38 and 10A. yellow p<0.01, red p<0.001.
(DOCX)

Table S6 **Chi-square values for individual CT pairs between cell types.** The chi-square p values are shown comparing WI38 to 10A for each individual CT pair in G1 and in S for differences in the percent of cells with only 1 interaction, ≥2 interactions, and when considering both = 1≥2 interactions together. Purple p<0.10, Green p<0.05, yellow p<0.01, red p<0.001.
(DOCX)

Dataset S1 Distance and volume measurements for CT and nuclei.
(XLSX)
