# Correction: Cell Type Specific Alterations in Interchromosomal Networks across the Cell Cycle

**DOI:** 10.1371/journal.pcbi.1004064

**Published:** 2014-12-09

**Authors:** The PLOS Computational Biology Staff

Figure 1 is incorrect. In Figure 1E, S phase calculations were deleted from the figure during formatting. The authors have provided a corrected version here. The figure legend remains correct.

**Figure 1:**
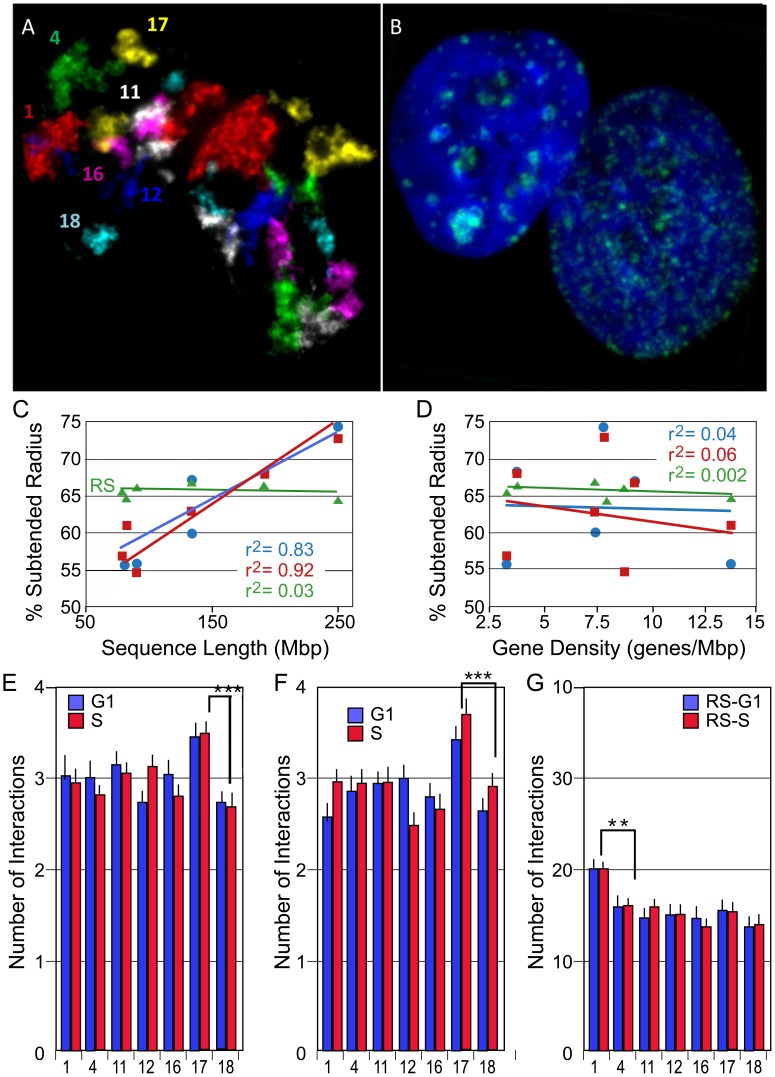
Multi-FISH labeling, radial positioning, and total interactions of CT during the cell cycle. 2-D projection images of: (A) 7 CT (1,4,11,12, 16,17,18) and (B) EdU labeled replication sites and DAPI staining in 10A cells. (C–D) Radial positioning- The percent subtended radii (the distance from the center of the nucleus to the CT center divided by the distance from the center of the nucleus through the CT center to the nuclear periphery in 10A cells) is displayed against chromosome sequence length (C) and gene density (D). Blue is G1, red is S, and green is random simulations. (E–F) Total pairwise CT interactions- The average number of CT within the subset of CT that each individual CT interacts with is shown for WI38 (E), 10A (F), and random simulations (G). Blue is G1 and red is S. All experimental values were significant higher than random simulations. CT17 interacts with a significantly greater number of CT than any other CT in the subset. CT1 is significantly higher than the other CT in random simulations. ***p<0.001.
