# Supplementary figures and images for: Cell Type Specific Alterations in Interchromosomal Networks across the Cell Cycle
Source: PLoS Comput Biol. 2014 Oct 2;10(10):e1003857. doi: 10.1371/journal.pcbi.1003857 (PMC4183423; doi:10.1371/journal.pcbi.1003857)

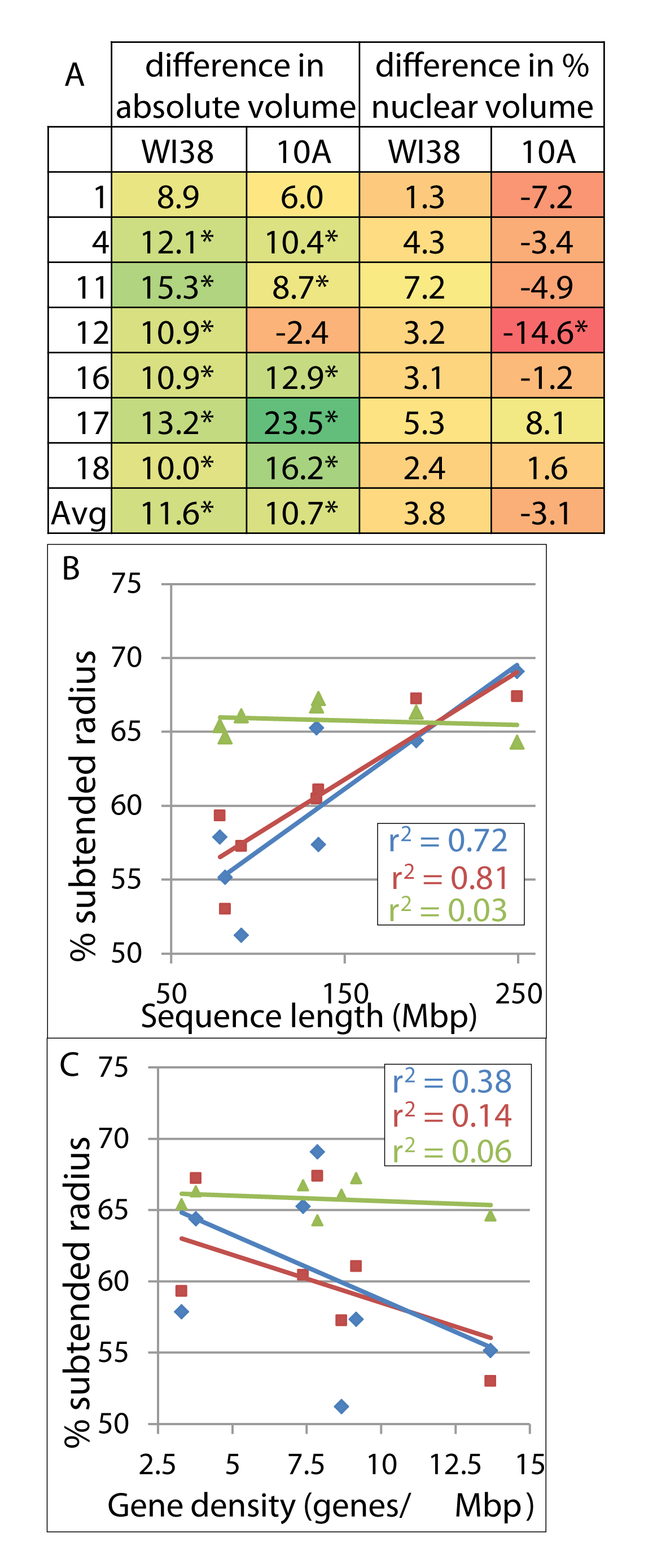

Supplement: Figure S1 — Nuclear volumes and radial positions of CT in the cell cycle. (A) The percent difference in CT absolute volumes from G1 to S are shown. The volume differences as a percent of the nucleus are also shown. (B) The relationship between radial positioning (% subtended radii) and sequence length (B) or gene density (C) in WI38 is shown. Blue is G1, Red is S, and green are random simulations. (TIF) [file pcbi.1003857.s001.tif]

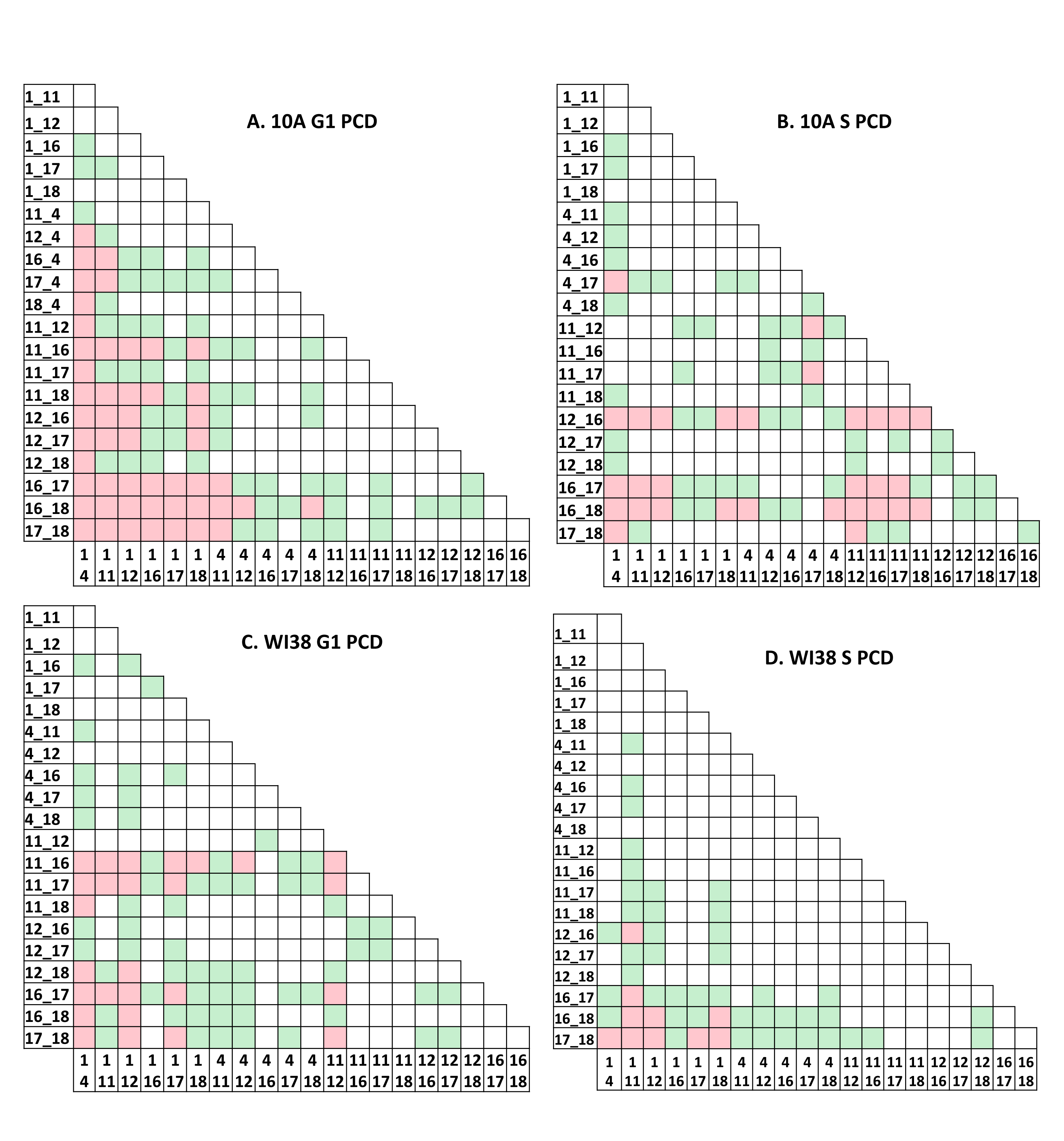

Supplement: Figure S2 — Comparison of experimental pairwise center distances (PCD) between all combinations. Experimental pairwise distances between CT centers were normalized to the major axis. Each position within the matrix represents a comparison between the pairwise CT centers in 10A G1 (A), 10A S (B), WI38 G1 (C), and WI38 S (D). Green squares represent p<0.05 and red p<0.001. (TIF) [file pcbi.1003857.s002.tif]

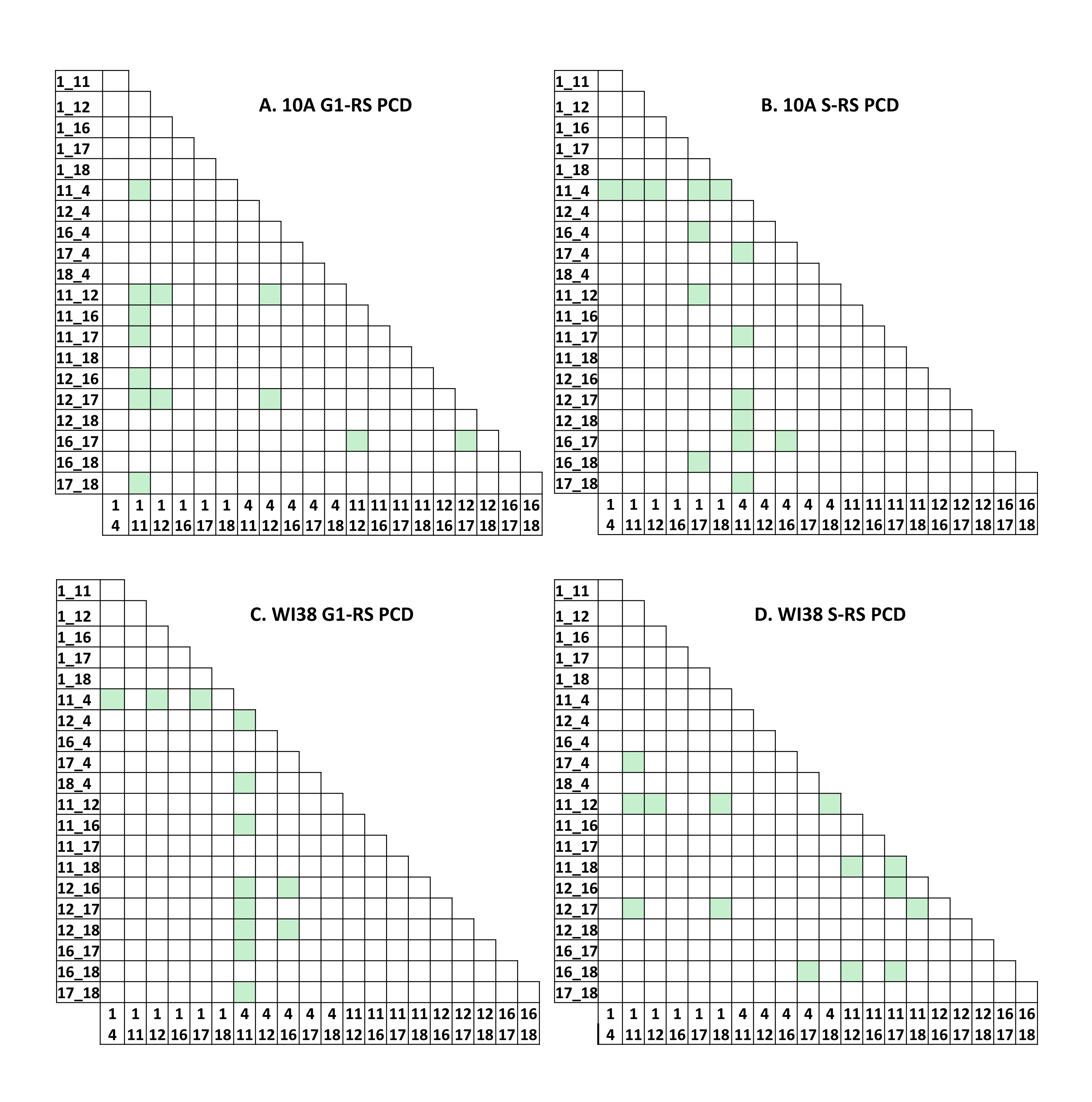

Supplement: Figure S3 — Comparison of pairwise center distances (PCD) between all combinations in random simulations. Each position within the matrix represents a ttest comparison between the normalized pairwise CT centers in random simulations of 10A G1 (A), 10A S (B), WI38 G1 (C), and WI38 S (D). Green squares represent p<0.05. (TIF) [file pcbi.1003857.s003.tif]

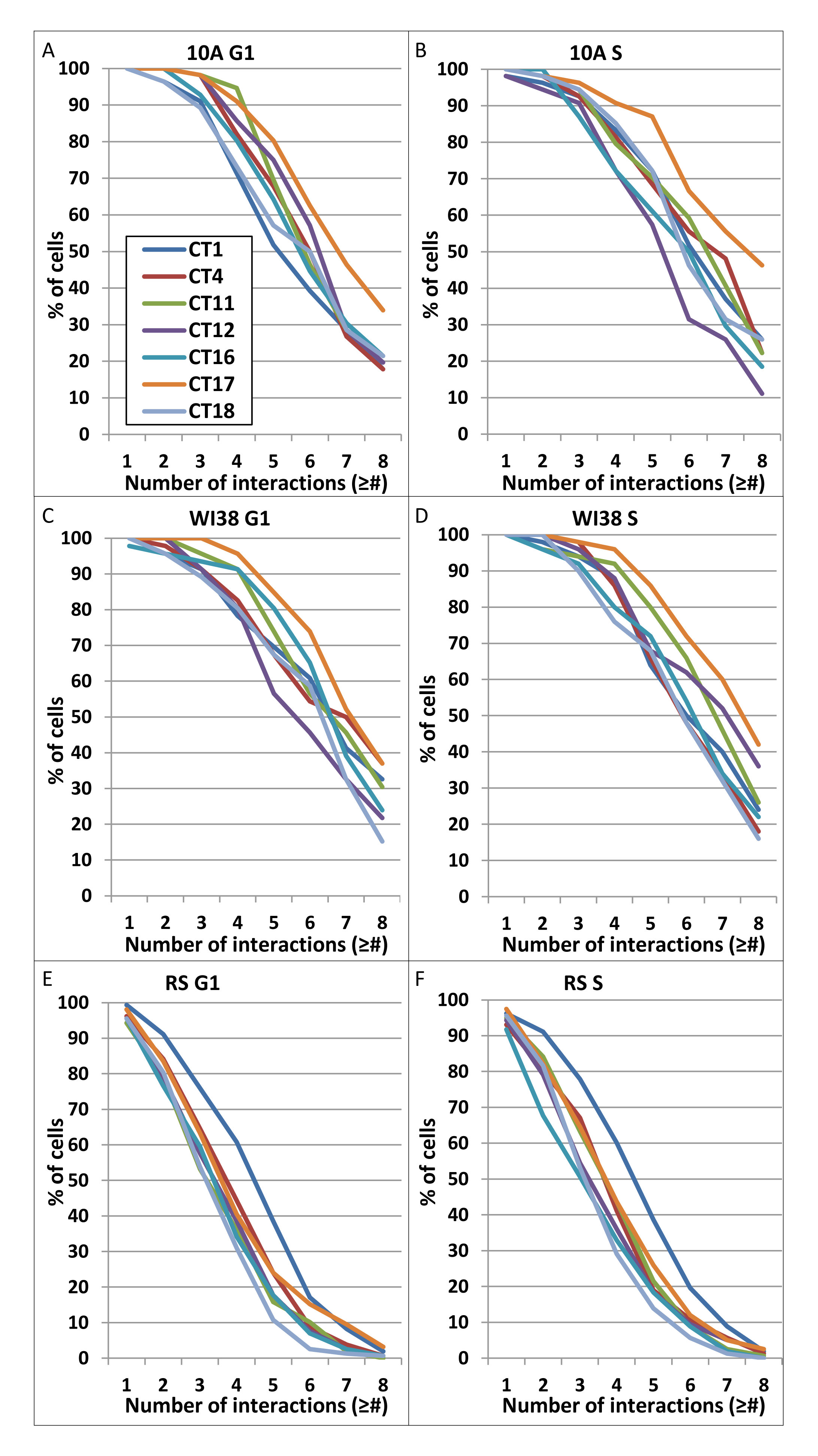

Supplement: Figure S4 — Distributions of total interactions between CT. The number of interactions between each CT pair and other CT studied was determined. The distributions of interactions (≥1, ≥2, etc.) for each CT are shown for 10A G1 (A), 10A S (B), WI38 G1 (C), and WI38 S (D), and random simulations of 10A G1 (E) or 10A S (F). (TIF) [file pcbi.1003857.s004.tif]

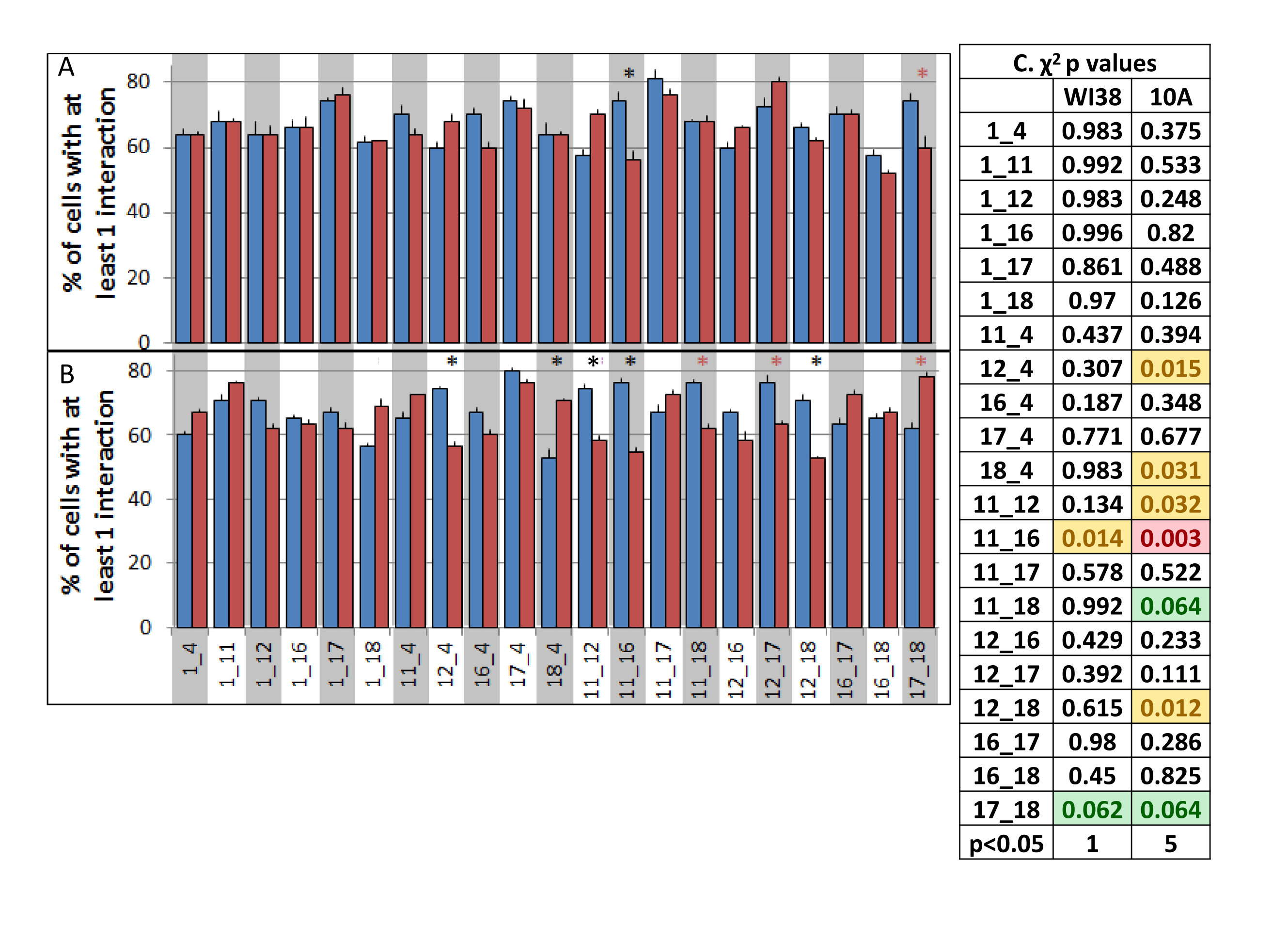

Supplement: Figure S5 — Interaction profiles of CT (≥1 interaction). The percent of cells with at least one interaction in WI38 (A) and 10A (B) are shown. Blue bars are G1 and red S. Chi square p values are shown for the difference between G1 and S (C). (TIF) [file pcbi.1003857.s005.tif]

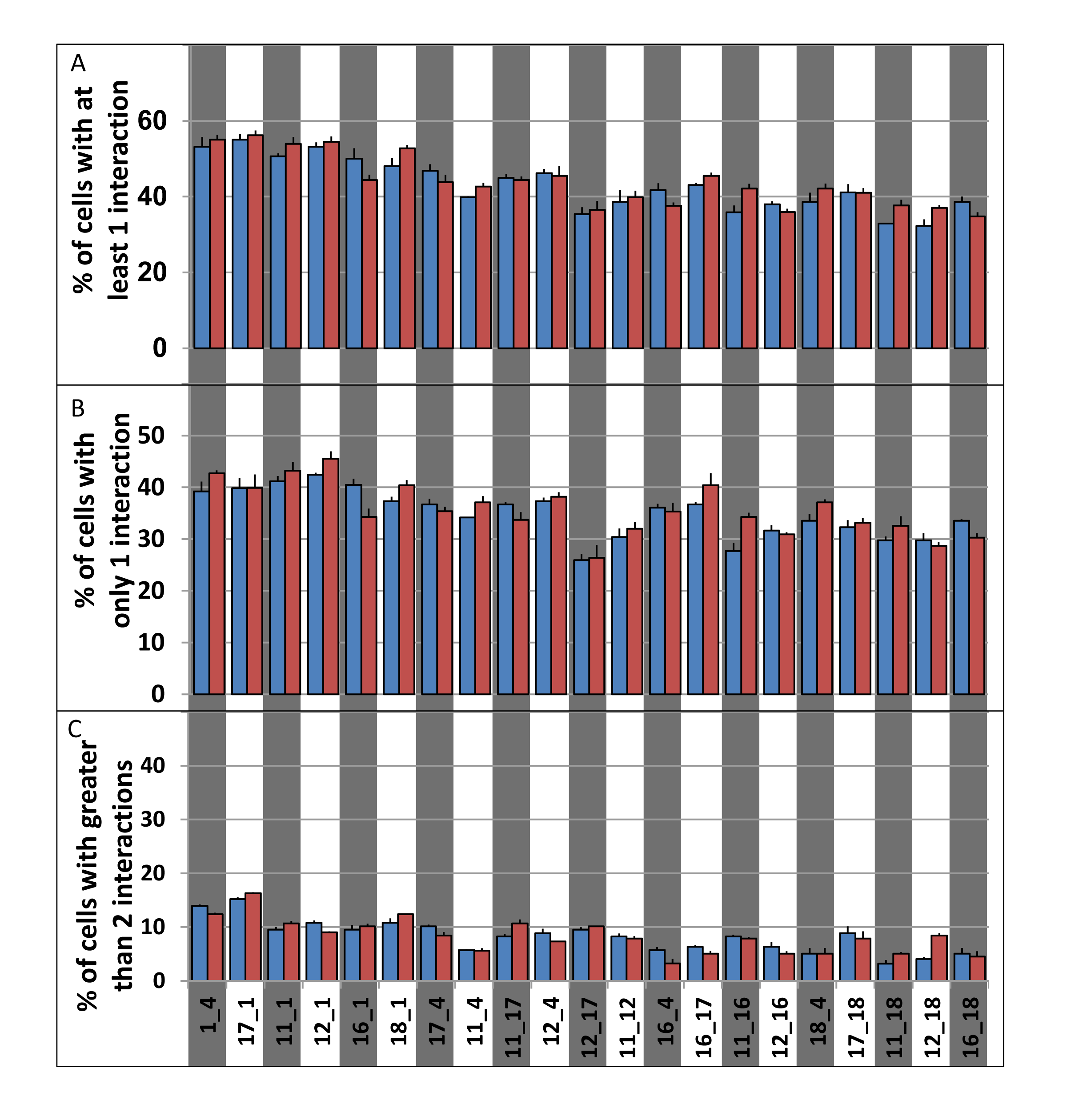

Supplement: Figure S6 — Interaction profile of CT in random simulations in G1 and S phase of WI38. Simulations were performed where CT of the same volume were grown asymmetrically simulating CT morphology inside the experimental nuclei (see materials and methods). The percent of cells with at least one interaction (A), only 1 interaction (B) and greater than 2 interactions (C) are shown. Blue bars are random simulations of G1 and red are random simulations of S. Error bars are SEM. (TIF) [file pcbi.1003857.s006.tif]

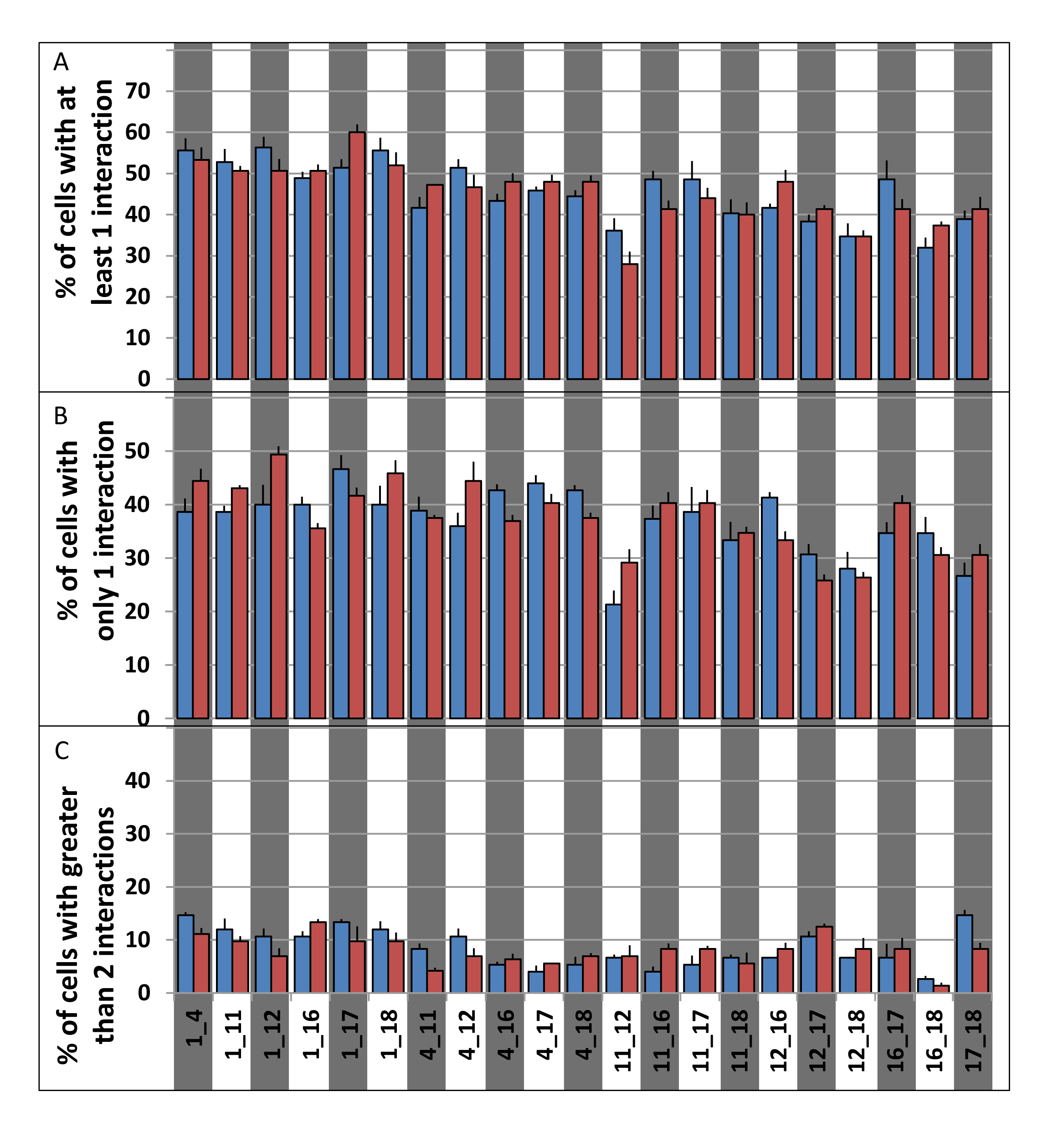

Supplement: Figure S7 — Interaction profile of CT in Random simulations in G1 and S phase of 10A. The percent of cells with at least one interaction (A), only 1 interaction (B) and greater than 2 interactions (C) are shown for random simulations using 10A G1 or S cells. Blue bars are random simulations of G1 and red are random simulations of S. Error bars are SEM. (TIF) [file pcbi.1003857.s007.tif]

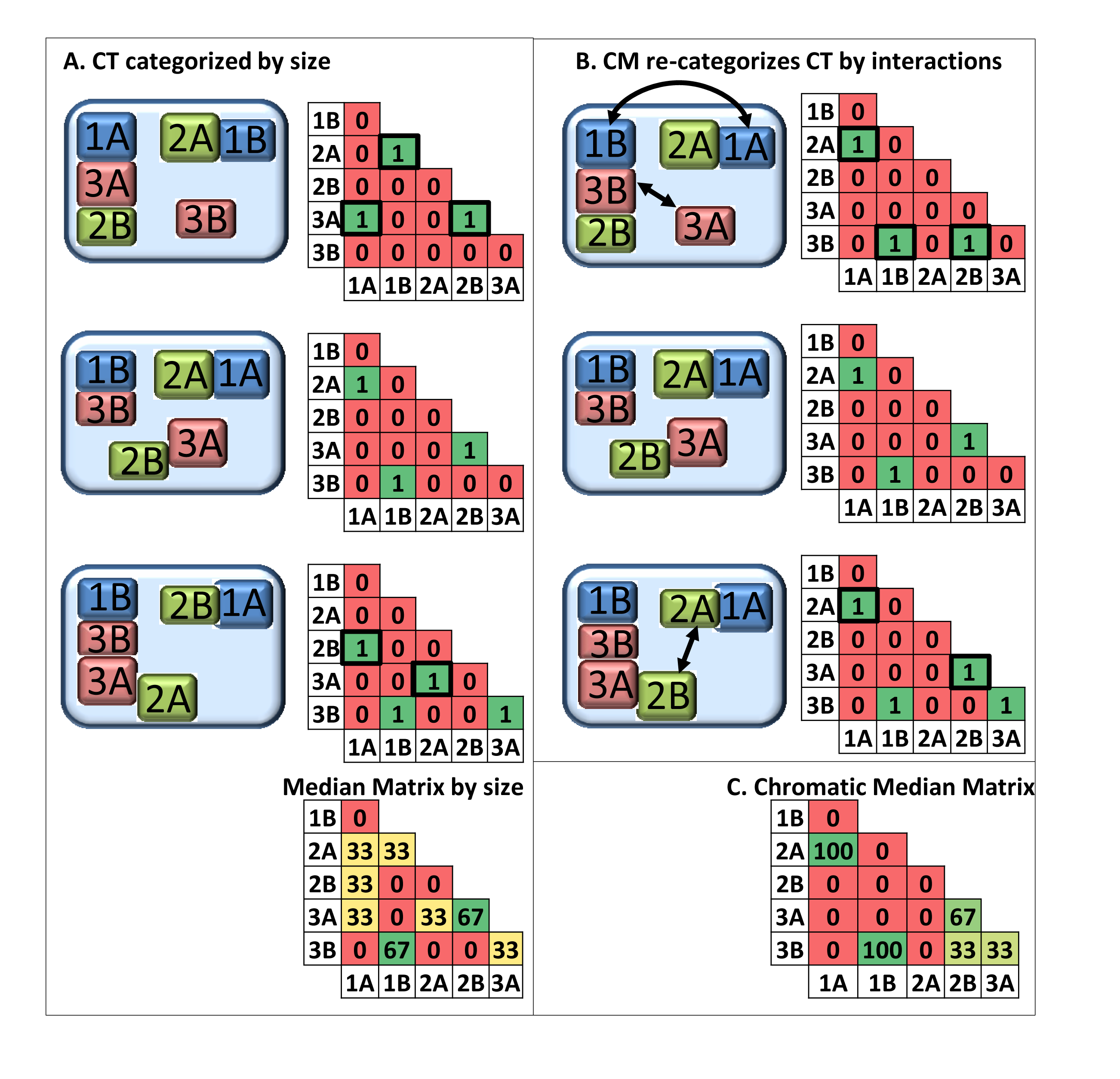

Supplement: Figure S8 — Schematic diagram of chromatic median analysis. The interactions between CT in each input nucleus are represented as a binary code in input matrices. The input into the chromatic median program is defined based upon CT volumes with the larger CT homolog termed “a” and the smaller “b” (A). After a permutation analysis that defines homolog a versus homolog b based upon each homologs interaction with other CT (B), the percent of cells with an interaction between all pairwise combinations is determined (C). CT homologs that are switched are indicated by double arrows and are outlined in bold in their corresponding matrices. The values are color coded on a color-scale from low (red) to high (green). (TIF) [file pcbi.1003857.s008.tif]

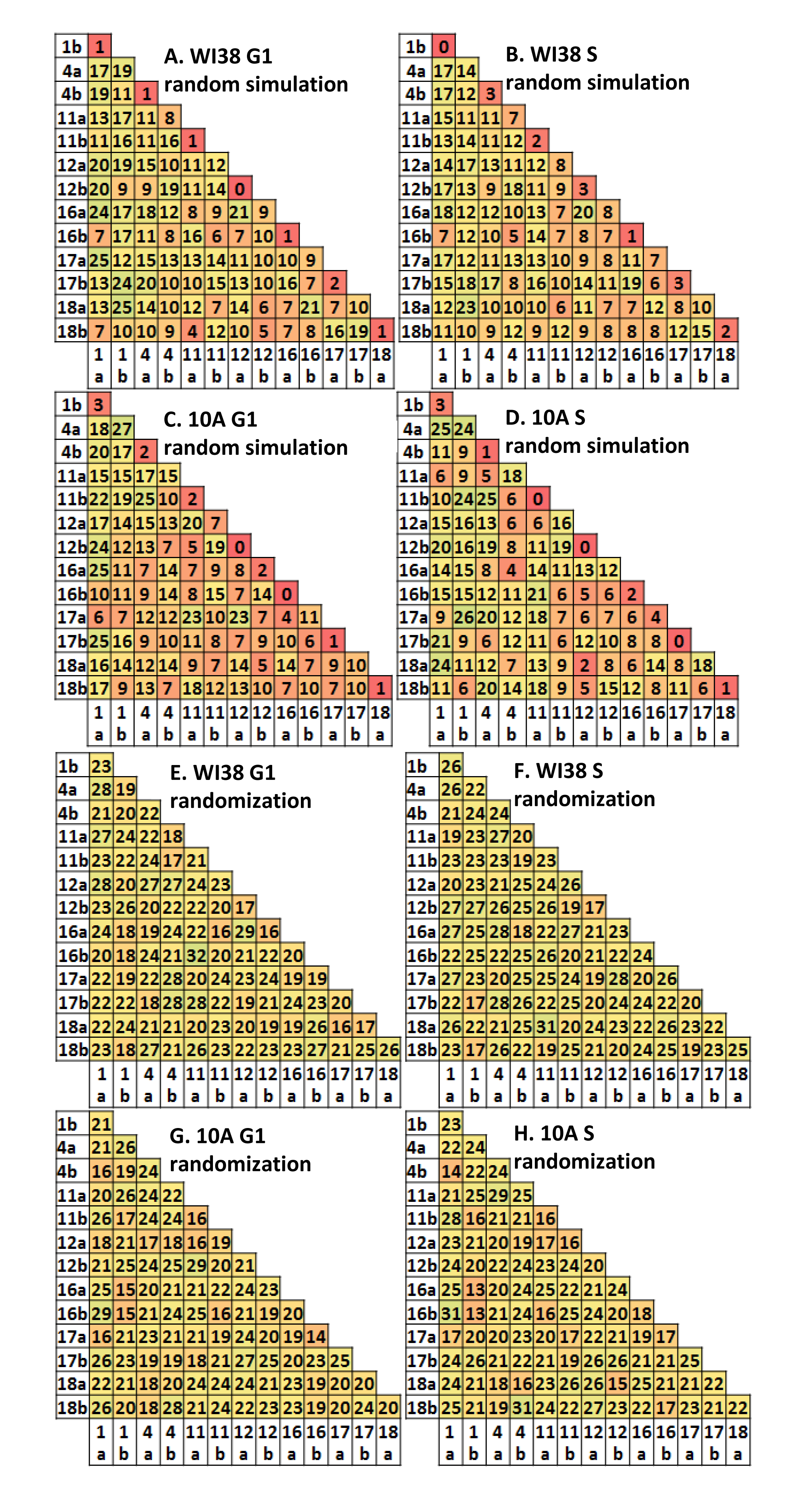

Supplement: Figure S9 — Chromatic median analysis of random simulations and randomizations in G1and S. The chromatic median algorithm determines correspondence between homologs across nuclei based upon which other CT it interacts. This algorithm determined a median matrix for CT interactions for random simulations that put CT of similar volume within the DAPI signal in WI38 (A–B) and 10A (C–D); in G1 (A, C) and S phase (B,D). Next we randomized the input matrices of the experimental input cells for WI38 G1 (E), WI38 S (F), 10A G1 (G), and 10A S (H). Each cell in the matrix represents the percent of input nuclei that have an interaction between those homologs. Values are color-coded on a color-scale from low (red) to high (green). (TIF) [file pcbi.1003857.s009.tif]
