# Supplementary material for: Cell Type Specific Alterations in Interchromosomal Networks across the Cell Cycle
Source: PLoS Comput Biol. 2014 Oct 2;10(10):e1003857. doi: 10.1371/journal.pcbi.1003857 (PMC4183423; doi:10.1371/journal.pcbi.1003857)
Supplement: Table S1 — Chi-square values comparing the overall patterns to uniform averages. The overall patterns were compared to their uniform averages. Chi-square p values are shown for experimental and random simulations. Green p<0.05, yellow p<0.01, red p<0.001. (DOCX) [file pcbi.1003857.s010.docx]

| overall pattern vs. uniform averages | | | | | | simulations vs. uniform averages | | | | |
| --- | --- | --- | --- | --- | --- | --- | --- | --- | --- | --- |
|  | W G1 | A G1 |  | W S | A S | W-RS G1 | A-RS G1 |  | W-RS S | A-RS S |
| ≥1 | 0.908 | 0.814 |  | 0.855 | 0.614 | 0.355 | 0.320 |  | 0.386 | 0.308 |
| =1 | 0.799 | 0.617 |  | 0.476 | 0.657 | 0.914 | 0.416 |  | 0.830 | 0.319 |
| ≥2 | 0.095 | 0.009 |  | 0.002 | <0.001 | 0.309 | 0.141 |  | 0.201 | 0.569 |
| =1&≥2 | 0.375 | 0.020 |  | 0.014 | 0.004 | 0.747 | 0.226 |  | 0.559 | 0.484 |
