# Supplementary material for: Cell Type Specific Alterations in Interchromosomal Networks across the Cell Cycle
Source: PLoS Comput Biol. 2014 Oct 2;10(10):e1003857. doi: 10.1371/journal.pcbi.1003857 (PMC4183423; doi:10.1371/journal.pcbi.1003857)
Supplement: Table S2 — Chi-square values comparing the overall patterns between G1 and S. The chi-square p values are shown comparing the overall patterns in G1 to S in 10A to WI38 and in random simulations for G1 versus S. Green p<0.05, yellow p<0.01, red p<0.001. (DOCX) [file pcbi.1003857.s011.docx]

| comparing cell cycle | | | cell cycle- simulations | | |
| --- | --- | --- | --- | --- | --- |
|  | G1vS W | G1vS A |  | G1vS W | G1vS S |
| ≥1 | 0.599 | <0.001 | ≥1 | 0.927 | 0.999 |
| 1 | <0.001 | <0.001 | 1 | 0.667 | 1.00 |
| ≥2 | <0.001 | <0.001 | ≥2 | 0.687 | 0.999 |
| 1&≥2 | <0.001 | <0.001 | 1&≥2 | 0.800 | 1.00 |
