# Supplementary material for: Cell Type Specific Alterations in Interchromosomal Networks across the Cell Cycle
Source: PLoS Comput Biol. 2014 Oct 2;10(10):e1003857. doi: 10.1371/journal.pcbi.1003857 (PMC4183423; doi:10.1371/journal.pcbi.1003857)
Supplement: Table S3 — Chi-square values of individual CT pairs across the cell cycle. The chi-square p values are shown comparing G1 to S for each individual CT pair in WI38 and in 10A for differences in the percent of cells with only 1 interaction, ≥2 interactions, and when considering both = 1≥2 interactions together. Purple p<0.10, Green p<0.05, yellow p<0.01, red p<0.001. (DOCX) [file pcbi.1003857.s012.docx]

| **WI38** | **W =1** | **W ≥2** | **=1&≥2** |  | **10A** | **A =1** | **A ≥2** | **=1&≥2** |
| --- | --- | --- | --- | --- | --- | --- | --- | --- |
| **1_4** | **0.007** | **<0.001** | **<0.001** |  | **1_4** | **0.070** | **0.003** | **0.001** |
| **1_11** | **0.694** | **0.669** | **0.845** |  | **1_11** | **1.000** | **0.280** | **0.459** |
| **1_12** | **0.238** | **0.087** | **0.115** |  | **1_12** | **<0.001** | **0.149** | **0.001** |
| **1_16** | **0.136** | **0.146** | **0.115** |  | **1_16** | **0.006** | **0.020** | **0.009** |
| **1_17** | **0.282** | **0.165** | **0.21** |  | **1_17** | **0.149** | **0.719** | **0.356** |
| **1_18** | **0.136** | **0.111** | **0.093** |  | **1_18** | **0.031** | **0.719** | **0.074** |
| **4_11** | **0.788** | **0.106** | **0.193** |  | **4_11** | **0.135** | **1.000** | **0.406** |
| **4_12** | **0.219** | **0.927** | **0.278** |  | **4_12** | **1.000** | **<0.001** | **<0.001** |
| **4_16** | **0.461** | **0.253** | **0.166** |  | **4_16** | **1.000** | **0.072** | **0.128** |
| **4_17** | **0.686** | **0.988** | **0.883** |  | **4_17** | **0.399** | **0.779** | **0.573** |
| **4_18** | **0.411** | **0.203** | **0.317** |  | **4_18** | **0.296** | **0.015** | **0.004** |
| **11_12** | **0.266** | **0.002** | **0.002** |  | **11_12** | **0.015** | **0.223** | **0.01** |
| **11_16** | **0.136** | **0.040** | **0.002** |  | **11_16** | **<0.001** | **0.243** | **<0.001** |
| **11_17** | **0.994** | **0.458** | **0.65** |  | **11_17** | **0.028** | **0.002** | **<0.001** |
| **11_18** | **0.788** | **0.736** | **0.911** |  | **11_18** | **0.042** | **0.223** | **0.032** |
| **12_16** | **0.496** | **0.669** | **0.529** |  | **12_16** | **0.340** | **<0.001** | **0.002** |
| **12_17** | **0.062** | **0.009** | **0.004** |  | **12_17** | **0.082** | **0.486** | **0.071** |
| **12_18** | **0.459** | **0.877** | **0.662** |  | **12_18** | **<0.001** | **0.653** | **0.001** |
| **16_17** | **0.470** | **0.461** | **0.587** |  | **16_17** | **0.122** | **0.002** | **0.002** |
| **16_18** | **0.969** | **0.228** | **0.363** |  | **16_18** | **0.744** | **1.000** | **0.932** |
| **17_18** | **<0.001** | **0.183** | **<0.001** |  | **17_18** | **0.191** | **0.102** | **0.027** |
| **total p<0.05** | **2** | **4** | **5** |  | **total p<0.05** | **8** | **7** | **13** |
