# Supplementary material for: Cell Type Specific Alterations in Interchromosomal Networks across the Cell Cycle
Source: PLoS Comput Biol. 2014 Oct 2;10(10):e1003857. doi: 10.1371/journal.pcbi.1003857 (PMC4183423; doi:10.1371/journal.pcbi.1003857)
Supplement: Table S4 — Homologous versus heterologous Interactions. The percent of total interactions for heterologous (4 possible per cell) and homologous (1 possible per cell) CT interactions were ranked from lowest to highest in G1 of WI38 or 10A. Heterologous interactions are normalized for the fact that there are 4 possible interactions. Homologous CT interactions are written in magenta. The values are color coded on a color-scale from low (red) to high (green). (DOCX) [file pcbi.1003857.s013.docx]

|  | **WI38 G1** | **WI38 S** |  |  | **10A G1** | **10A S** |  |  | **WI38 G1** | **10A G1** |  |  | **WI38 S** | **10A S** |
| --- | --- | --- | --- | --- | --- | --- | --- | --- | --- | --- | --- | --- | --- | --- |
| **4_4** | **17.39** | **12.00** |  | **4_4** | **8.93** | **11.11** |  | **4_4** | **17.39** | **8.93** |  | **1_1** | **10.00** | **7.41** |
| **18_18** | **17.39** | **14.00** |  | **16_16** | **12.50** | **9.26** |  | **18_18** | **17.39** | **21.43** |  | **4_4** | **12.00** | **11.11** |
| **11_11** | **19.57** | **22.00** |  | **4_18** | **16.96** | **25.91** |  | **11_11** | **19.57** | **17.86** |  | **18_18** | **14.00** | **9.26** |
| **16_16** | **19.57** | **22.00** |  | **11_11** | **17.86** | **14.81** |  | **16_16** | **19.57** | **12.50** |  | **12_12** | **20.00** | **11.11** |
| **17_17** | **19.57** | **22.00** |  | **12_12** | **17.86** | **11.11** |  | **17_17** | **19.57** | **23.21** |  | **16_18** | **20.65** | **26.36** |
| **11_12** | **20.11** | **30.98** |  | **1_16** | **19.20** | **22.73** |  | **11_12** | **20.11** | **25.89** |  | **11_11** | **22.00** | **14.81** |
| **12_18** | **20.65** | **23.37** |  | **1_1** | **19.64** | **11.11** |  | **12_18** | **20.65** | **22.77** |  | **16_16** | **22.00** | **9.26** |
| **1_18** | **21.20** | **26.09** |  | **16_17** | **20.09** | **28.18** |  | **1_18** | **21.20** | **20.54** |  | **17_17** | **22.00** | **12.96** |
| **16_18** | **21.20** | **20.65** |  | **1_4** | **20.54** | **26.82** |  | **16_18** | **21.20** | **24.55** |  | **4_18** | **22.28** | **25.91** |
| **4_12** | **21.74** | **26.63** |  | **1_18** | **20.54** | **24.55** |  | **4_12** | **21.74** | **27.23** |  | **11_16** | **22.83** | **20.91** |
| **12_12** | **21.74** | **20.00** |  | **17_18** | **20.54** | **27.73** |  | **12_12** | **21.74** | **17.86** |  | **12_18** | **23.37** | **19.09** |
| **12_16** | **22.28** | **27.17** |  | **18_18** | **21.43** | **14.81** |  | **12_16** | **22.28** | **24.55** |  | **1_12** | **24.46** | **22.27** |
| **11_18** | **23.37** | **26.63** |  | **1_12** | **22.32** | **22.27** |  | **11_18** | **23.37** | **26.79** |  | **1_4** | **25.00** | **26.82** |
| **1_12** | **23.91** | **24.46** |  | **4_11** | **22.32** | **25.00** |  | **1_12** | **23.91** | **22.32** |  | **4_11** | **25.00** | **25.00** |
| **1_1** | **23.91** | **16.00** |  | **1_11** | **22.77** | **27.27** |  | **1_1** | **23.91** | **19.64** |  | **4_16** | **25.54** | **19.09** |
| **4_18** | **24.46** | **22.28** |  | **12_18** | **22.77** | **19.09** |  | **4_18** | **24.46** | **16.96** |  | **1_18** | **26.09** | **24.55** |
| **1_16** | **25.54** | **27.72** |  | **11_17** | **23.21** | **32.27** |  | **1_16** | **25.54** | **19.20** |  | **1_11** | **26.63** | **27.27** |
| **4_11** | **25.54** | **25.00** |  | **17_17** | **23.21** | 18.51 |  | **4_11** | **25.54** | **22.32** |  | **4_12** | **26.63** | **17.73** |
| **4_16** | **26.09** | **25.54** |  | **1_17** | **24.11** | **23.64** |  | **4_16** | **26.09** | **24.11** |  | **11_18** | **26.63** | **21.36** |
| **17_18** | **26.09** | **28.80** |  | **4_16** | **24.11** | **19.09** |  | **17_18** | **26.09** | **20.54** |  | **12_16** | **27.17** | **18.64** |
| **1_4** | **26.63** | **25.00** |  | **12_16** | **24.55** | **18.64** |  | **1_4** | **26.63** | **20.54** |  | **1_16** | **27.72** | **22.73** |
| **1_11** | **27.17** | **26.63** |  | **16_18** | **24.55** | **26.36** |  | **1_11** | **27.17** | **22.77** |  | **4_17** | **28.80** | **32.27** |
| **1_17** | **27.17** | **31.52** |  | **11_12** | **25.89** | **21.36** |  | **1_17** | **27.17** | **24.11** |  | **17_18** | **28.80** | **27.73** |
| **11_16** | **27.17** | **22.83** |  | **11_18** | **26.79** | **21.36** |  | **11_16** | **27.17** | **27.23** |  | **16_17** | **29.35** | **28.18** |
| **4_17** | **27.72** | **28.80** |  | **4_12** | **27.23** | **17.73** |  | **4_17** | **27.72** | **32.14** |  | **11_12** | **30.98** | **21.36** |
| **12_17** | **27.72** | **38.04** |  | **11_16** | **27.23** | **20.91** |  | **12_17** | **27.72** | **28.13** |  | **1_17** | **31.52** | **23.64** |
| **16_17** | **30.98** | **29.35** |  | **12_17** | **28.13** | **24.55** |  | **16_17** | **30.98** | **20.09** |  | **11_17** | **34.78** | **32.27** |
| **11_17** | **34.78** | **34.78** |  | **4_17** | **32.14** | **32.27** |  | **11_17** | **34.78** | **23.21** |  | **12_17** | **38.04** | **24.55** |
