# Supplementary material for: Cell Type Specific Alterations in Interchromosomal Networks across the Cell Cycle
Source: PLoS Comput Biol. 2014 Oct 2;10(10):e1003857. doi: 10.1371/journal.pcbi.1003857 (PMC4183423; doi:10.1371/journal.pcbi.1003857)
Supplement: Table S5 — Chi-square values comparing the overall patterns between cell types within G1 or S. The chi-square p values are shown comparing the overall patterns in WI38 to 10A in G1 and in S and comparing random simulations of WI38 and 10A. yellow p<0.01, red p<0.001. (DOCX) [file pcbi.1003857.s014.docx]

| comparing cell types | | | cell type- simulations | | |
| --- | --- | --- | --- | --- | --- |
|  | WvA G1 | WvA S |  | WvA G1 | WvA S |
| ≥1 | 0.349 | 0.122 | ≥1 | 0.618 | 0.815 |
| 1 | 0.002 | 0.002 | 1 | 0.39 | 0.452 |
| ≥2 | <0.001 | <0.001 | ≥2 | 0.174 | 0.055 |
| 1&≥2 | <0.001 | <0.001 | 1&≥2 | 0.244 | 0.133 |
