# Supplementary material for: Cell Type Specific Alterations in Interchromosomal Networks across the Cell Cycle
Source: PLoS Comput Biol. 2014 Oct 2;10(10):e1003857. doi: 10.1371/journal.pcbi.1003857 (PMC4183423; doi:10.1371/journal.pcbi.1003857)
Supplement: Table S6 — Chi-square values for individual CT pairs between cell types. The chi-square p values are shown comparing WI38 to 10A for each individual CT pair in G1 and in S for differences in the percent of cells with only 1 interaction, ≥2 interactions, and when considering both = 1≥2 interactions together. Purple p<0.10, Green p<0.05, yellow p<0.01, red p<0.001. (DOCX) [file pcbi.1003857.s015.docx]

|  | **G1 =1** | **G1 ≥2** | **=1&≥2** |  |  | **S =1** | **S ≥2** | **=1&≥2** |
| --- | --- | --- | --- | --- | --- | --- | --- | --- |
| **1_4** | **0.007** | **0.003** | **<0.001** |  | **1_4** | **0.026** | **<0.001** | **<0.001** |
| **1_11** | **0.099** | **0.145** | **0.024** |  | **1_11** | **0.036** | **0.407** | **0.043** |
| **1_12** | **0.001** | **0.072** | **<0.001** |  | **1_12** | **0.250** | **0.223** | **0.096** |
| **1_16** | **0.026** | **0.004** | **<0.001** |  | **1_16** | **0.083** | **0.037** | **0.003** |
| **1_17** | **0.643** | **0.444** | **0.339** |  | **1_17** | **0.565** | **0.079** | **0.03** |
| **1_18** | **0.226** | **0.609** | **0.153** |  | **1_18** | **0.040** | **0.407** | **0.048** |
| **11_4** | **0.569** | **0.143** | **0.074** |  | **11_4** | **0.151** | **0.941** | **0.194** |
| **12_4** | **0.183** | **0.154** | **0.082** |  | **12_4** | **0.978** | **0.025** | **0.003** |
| **16_4** | **0.214** | **0.074** | **0.015** |  | **16_4** | **0.040** | **0.028** | **<0.001** |
| **17_4** | **0.696** | **0.152** | **0.18** |  | **17_4** | **0.388** | **0.083** | **0.075** |
| **18_4** | **0.724** | **0.067** | **0.019** |  | **18_4** | **0.727** | **0.029** | **0.07** |
| **11_12** | **0.081** | **0.150** | **0.047** |  | **11_12** | **0.274** | **0.004** | **<0.001** |
| **11_16** | **0.173** | **0.246** | **0.073** |  | **11_16** | **0.898** | **0.656** | **0.628** |
| **11_17** | **0.100** | **<0.001** | **<0.001** |  | **11_17** | **0.400** | **0.801** | **0.346** |
| **11_18** | **0.042** | **0.427** | **0.049** |  | **11_18** | **0.774** | **0.131** | **0.07** |
| **12_16** | **0.152** | **0.941** | **0.2** |  | **12_16** | **0.203** | **0.005** | **<0.001** |
| **12_17** | **0.656** | **0.837** | **0.635** |  | **12_17** | **0.440** | **0.003** | **<0.001** |
| **12_18** | **0.229** | **0.502** | **0.185** |  | **12_18** | **0.565** | **0.229** | **0.129** |
| **16_17** | **0.001** | **<0.001** | **<0.001** |  | **16_17** | **0.473** | **0.808** | **0.471** |
| **16_18** | **0.383** | **0.537** | **0.318** |  | **16_18** | **0.245** | **0.058** | **0.055** |
| **17_18** | **0.484** | **0.144** | **0.063** |  | **17_18** | **<0.001** | **0.250** | **<0.001** |
| **total p<0.05** | **5** | **4** | **10** |  | **total p<0.05** | **5** | **8** | **11** |
